# Development of an open-source software for isomer enumeration

**DOI:** 10.1186/s13321-022-00677-6

**Published:** 2023-01-22

**Authors:** Salomé R. Rieder, Marina P. Oliveira, Sereina Riniker, Philippe H. Hünenberger

**Affiliations:** Laboratorium für Physikalische Chemie, ETH Zürich, Vladimir-Prelog-Weg 2, 8093 Zürich, Switzerland

**Keywords:** Isomer enumeration, Graph isomorphism, Stereoisomerism

## Abstract

This article documents *enu*, a freely-downloadable, open-source and stand-alone program written in C++ for the enumeration of the constitutional isomers and stereoisomers of a molecular formula. The program relies on graph theory to enumerate all the constitutional isomers of a given formula on the basis of their canonical adjacency matrix. The stereoisomers of a given constitutional isomer are enumerated as well, on the basis of the automorphism group of this matrix. The isomer list is then reported in the form of canonical SMILES strings within files in XML format. The specification of the molecule family of interest is very flexible and the code is optimized for computational efficiency. The algorithms and implementations underlying *enu* are described, and simple illustrative applications are presented. The *enu* code is freely available on GitHub at https://github.com/csms-ethz/CombiFF.

**Supplementary Information:**

The online version contains supplementary material available at 10.1186/s13321-022-00677-6.

## Introduction

Chemistry is the science of molecular transformations, i.e., the recombination of sets of atoms in different molecules. Therefore, the concepts of molecular formula (atom content), structure (connectivity and stereochemistry), and geometry (conformation) are central to the field of chemistry.

Except for the smallest compounds, there generally exist many molecular structures compatible with a given formula. The corresponding molecules are referred to as *isomers*, and their number typically increases exponentially with the number of atoms in the molecule. Among these isomers, one may further distinguish between *constitutional isomers* and *stereoisomers*. Constitutional isomers differ exclusively in terms of the connectivity of the atoms, disregarding any spatial considerations. Given a specific constitutional isomer, the associated stereoisomers typically differ by the *chirality* or the *cis-trans isomery* of specific groups in the molecule. These spatial differences are ascribed to structure (topology) rather than to geometry (conformation) because the interconversion between stereoisomers does not occur spontaneously under usual conditions. As a result, the individual stereoisomers can be isolated, and their physicochemical properties are generally distinct.

The determination of isomer sets has been of interest in the fields of chemistry, mathematics, and computer science for a long time [1, 2]. Isomer counting is in itself already a very challenging mathematical problem in the field of graph theory, that has been addressed since nearly a century [3]. In view of the large isomer counts for all but the smallest compounds, the explicit enumeration of the isomers of a given molecular formula was essentially impossible before the development of sufficiently powerful computers. In this context, one may mention the pioneering DENDRAL project, going back to the 1960s [1, 4]. Since then, many efficient algorithms have been developed for performing isomer enumeration in an efficient way [5–13]. A historic overview can be found in the recent article by Yirik et al. [14].

From a fundamental point of view, isomer counting and enumeration are important tools to improve our knowledge of chemical space [11], and to analyze the effective coverage of chemical databases in terms of this space [1]. Isomer enumeration can also be used as a starting point for structure elucidation (by generating structures fulfilling certain restrictions obtained from spectroscopy) and virtual screening (by generating candidate structures) [1].

Recently, our group has introduced a new scheme called CombiFF to design classical force fields for molecular simulation [15, 16], in which isomer enumeration plays a central role. More specifically, CombiFF performs the automated calibration of force-field parameters against experimental condensed-phase data, considering entire classes of organic molecules constructed using a fragment library *via* combinatorial isomer enumeration. The main steps of the scheme are: (i) definition of a molecule family; (ii) enumeration of all isomers; (iii) query for experimental data; (iv) automatic construction of the molecular topologies by fragment assembly; (v) iterative refinement of the force-field parameters considering the entire family.

The goal of the present article is to document the isomer enumerator of the CombiFF workflow, a C++ program written from scratch by the first author and called *enu*. Although the motivation for the development of *enu* was the CombiFF scheme, the program is an open-source and stand-alone software that can be used and further developed independently of CombiFF for any other purpose in cheminformatics.

The main features of the *enu* program are the following:

1. The constitutional isomers of a given molecular formula are enumerated on the basis of their adjacency matrix, given the constraint of fixed valences for the different atom types and the application of a canonicalization by lexicographical matrix ordering.
2. The stereoisomers of a given constitutional isomer are enumerated on the basis of the automorphism group of the adjacency matrix.
3. The generated constitutional isomers and stereoisomers are reported in the form of canonical Simplified Molecular-Input Line-Entry System (SMILES) [17] strings within files following an Extensible Markup Language (XML) format.
4. The specification of the molecule family of interest is very flexible, including count ranges for the atoms in the molecular formula, selectors for specified substructures, and values of basic properties such as the number of cycles, unsaturations or multiple bonds.
5. After the initial implementation, care was taken to improve the computational efficiency of the C++ code, which is essential due to the combinatorial explosion of isomer counts with the number of atoms.
6. The code is freely available on GitHub at https://github.com/csms-ethz/CombiFF. The version of the code at the time of writing is available at https://github.com/csms-ethz/CombiFF/releases/tag/v1.0-beta.

The algorithm used in *enu* for the enumeration of constitutional isomers is largely inspired from the PhD thesis of R. Grund at the University of Bayreuth in 1994 [9], which is also the approach underlying the structure generator MOLGEN [1, 10, 18]. The enumeration of stereoisomers based on the automorphism group of the adjacency matrix, on the other hand, was developed independently by us. The generation of canonical SMILES strings is based on the works of Weininger et al. [19, 20] and Schneider et al. [21].

The present article describes the algorithms and features of *enu* in terms of the six points above. The main text focuses principally on the novel features of *enu*, namely the stereoisomer generation and the implementation and features of *enu*. More details on the other points (including the generation and canonicalization of constitutional isomers) are provided in the Additional file 1.

## Implementation

This section consists of five parts. First, it provides an overview of the basic principles underlying (molecular) graph theory. Second, it describes the enumeration algorithm for constitutional isomers developed by Grund. Third, it briefly explains the concept of canonical SMILES strings. Fourth, it describes the procedure developed here for the enumeration of stereoisomers. And fifth, it illustrates the implementation and practical features of the program *enu*.

### Graph theory

The isomer enumerator relies on graph theory. This discipline goes back to the first half of the 18th century when the Swiss mathematician Leonhard Euler published his famous article on the *Problem of the Königsberg Bridges* [22]. Since then, graph theory has become increasingly relevant, with applications in fields such as social sciences, economics, electrical and industrial engineering, as well as all branches of the natural sciences, namely physics, chemistry, and biology [23].

#### Molecular graphs

A molecular graph is a connected labeled multigraph, i.e., a graph in which there exists a path from each node to every other node, the nodes are labeled, and there can be multiple edges between two nodes. The vertices represent atoms and the edges account for covalent bonds between the atoms [24]. The graph describes the topology of a molecule, but does not provide any information on its geometry.

**Fig. 1:**
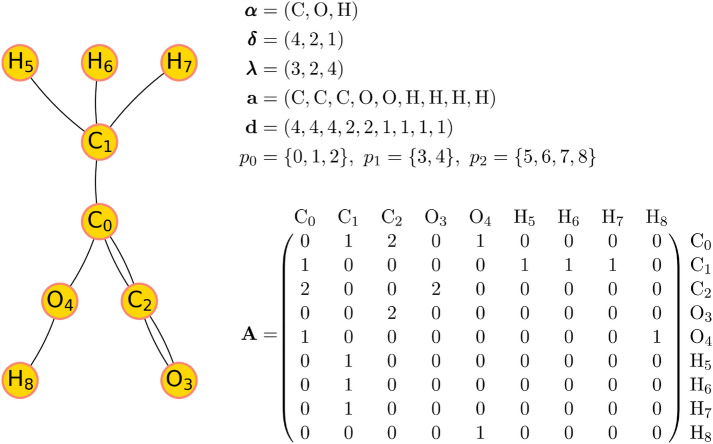
Illustrative example for a molecular graph. The graph corresponds to an isomer of the chemical formula $\hbox {C}_{3}\hbox {O}_{2}\hbox {H}_{4}$ with the label vector $\pmb \alpha$, the valence vector $\pmb \delta$, the partition vector $\pmb \lambda$, the atom vector $\pmb a$, the degree vector $\pmb d$, the partitions $p_0$, $p_1$ and $p_2$, and the adjacency matrix $\pmb A$

A molecular graph can be described by the combination of a *label vector* $\pmb \alpha$, a *valence vector* $\pmb \delta$, a *partition vector* $\pmb \lambda$, and a symmetric *adjacency matrix* $\pmb A\in {\mathbb {N}}_0^{+ \, N\times N}$ [9]. An example is provided in Fig. 1 and the terminology is explained in more detail in Additional file 1: Sec. S1.1. The combination of the label vector (atom-type names) and partition vector (number of atoms of a given type) provides the molecular formula. The valence vector contains the fixed valences of the atom types listed in the label vector. A matrix element $A_{i,j}$ of $\pmb A$ describes the order of the bond possibly connecting the atom at position *i* to the atom at position *j* in the atom vector (or is set to zero in the absence of a bond).

For a given choice of $\pmb \alpha$, $\pmb \delta$, and $\pmb \lambda$ (i.e., of a molecular formula and of atom-type valences), the specification of an adjacency matrix $\pmb A$ (i.e., of a covalent connectivity between the atoms) defines a unique labeled molecular graph. However, since the atoms of a common type in a molecule are physically indistinguishable, two labeled graphs that are directly related by a permutation in the indices of these atoms actually describe the same molecule (merely with a different atom numbering). In other words, for a given choice of $\pmb \alpha$, $\pmb \delta$, and $\pmb \lambda$, the same molecule can generally be represented by many different adjacency matrices $\pmb A$. This observation is fundamentally important to the problem of isomer enumeration. It is known as (molecular) graph isomorphism, and explained in more detail in Additional file 1: Sec. S1.2.

*Adjacency Matrix Canonicity* In order to have a unique representation of a molecular topology in the form of a labeled multigraph, a *lexicographical ordering* can be used as canonicity criterion for the adjacency matrix. An adjacency matrix $\pmb A$ is lexicographically larger than an adjacency matrix $\pmb A'$ (noted $\pmb A> \pmb A'$) provided that [9]

1 $$\begin{aligned} \exists i_0, j_0: \, \left( A_{i,j} = A'_{i,j} \, \, \, \forall \, (i,j) < (i_0, j_0)\right) \, \, \, \wedge \, \, \, \left( A_{i_0,j_0} > A'_{i_0, j_0}\right) \, , \end{aligned}$$

with the definition [9]

2 $$\begin{aligned} (i,j)< (k,l) \, \, \, \Leftrightarrow \, \, \, (i< k) \, \, \vee \, \, (i=k \, \wedge \, j < l) \, . \end{aligned}$$

In plain words, when the two matrices are read row-by-row from the top left to the bottom right, the first difference encountered determines the lexicographical ordering. The canonical adjacency matrix used to represent a molecule is then defined as the lexicographically largest among all possible adjacency matrices, which in turn defines a canonical labeling of the atoms in the molecular graph. Note that for a unique representation of molecules, the canonicity criterion for $\pmb A$ must be accompanied by a canonicity criterion for the ordering of the atom types in the vector $\pmb \alpha$. More details on canonicity can be found in Additional file 1: Sec. S1.2.

### Enumeration of constitutional isomers

The following sections present the algorithm that is used in *enu*. It is based on the PhD thesis of R. Grund [9], which proposes a solution to both the problem of finding all possible adjacency matrices as well as testing these matrices for canonicity.

Enumerating all the unique constitutional isomers of a given molecular formula amounts to finding all the canonical adjacency matrices associated with this formula. This could be achieved in a brute-force way by exhaustively enumerating all possible adjacency matrices compatible with the given molecular formula and the fixed valences of the different atom types, and filtering out those that are not canonical. The algorithm implemented in *enu* is based on this principle, but relies on an effective pruning mechanism that drastically limits the number of adjacency matrices to be generated and tested for canonicity, leading to far superior performance compared with a brute-force approach. A comparison between the optimized code and a brute-force approach is provided in Additional file 1: Sec. S1.7, see Table S1 and Figure S5.

#### Orderly enumeration

**Fig. 2:**
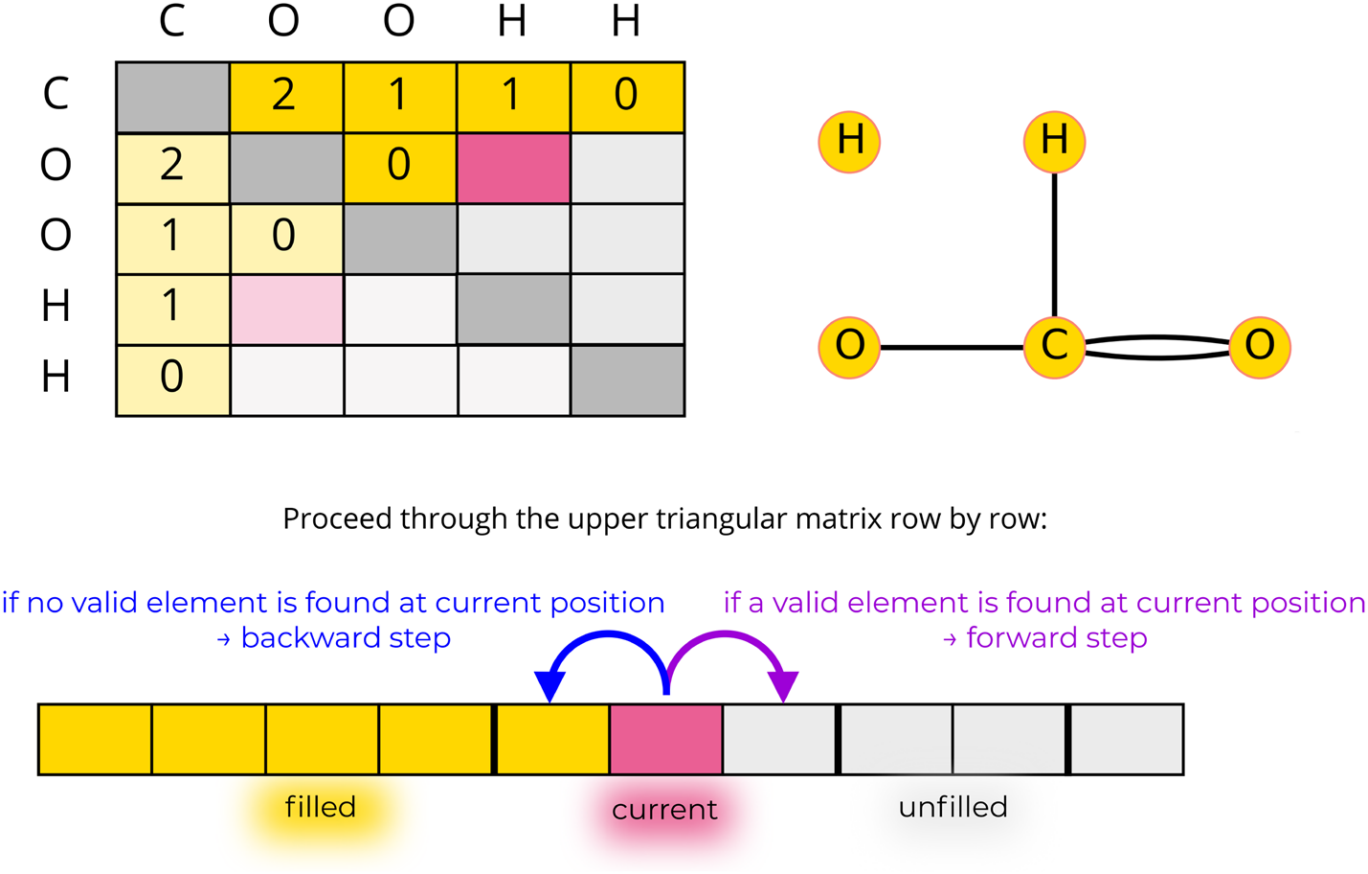
Illustrative example of the orderly enumeration algorithm. Top: A step of the filling algorithm for $\pmb \alpha =$(C,O,H), $\pmb \delta = (4,2,1)$, and $\pmb \lambda =(1,2,2)$ with an intermediate adjacency matrix and the corresponding molecular graph. Bottom: Schematic illustration describing how the algorithm proceeds through the adjacency matrix

The first task to be performed is the systematic construction of possible adjacency matrices for a given choice of the vectors $\pmb \alpha$, $\pmb \delta$, and $\pmb \lambda$. The algorithm of Grund [9] proceeds by creating these matrices in lexicographically decreasing order (Fig. 2). Note that this principle of orderly generation was proposed earlier by Read and Faradzev [25–27]. Since the adjacency matrix is symmetric and has only zeros along its diagonal, the algorithm only needs to find valid entries for the upper triangle of the matrix. Starting with an empty adjacency matrix, it proceeds through the matrix from the top left element to the bottom right one (with the line number as a primary index and, within each line, the column number as a secondary index), and fills it using two main subroutines. This filling order is particularly convenient, as it matches that in which the elements are checked to determine if a matrix is lexicographically larger or smaller than another one. In the *forward step*, the current matrix position is incremented and the maximum possible entry for the new position is determined based on the specified atom valences and the bonds already listed in the matrix. If a compatible value is found, the matrix element at the current position is filled, and another forward step is called. Otherwise, the matrix is not amenable to completion and a *backward step* is performed. The current matrix position is decremented and it is checked whether the matrix element at the new position can be decreased by one. If this is possible, the algorithm continues with a forward step. Otherwise it continues with another backward step. The two routines are outlined in the Additional file 1: Sec. S1.3.

#### Connectivity test

While the filling algorithm generates all possible adjacency matrices compatible with the valences of the different atoms in decreasing lexicographical order, it does not guarantee that these adjacency matrices describe a connected graph [9]. Therefore, a potential adjacency matrix has to be tested for connectivity to ensure that it is a viable isomer of the given molecular formula (rather than a collection of two or more molecules). The connectivity test implemented in *enu* is an adapted depth-first search [28] of the graph, as described in Additional file 1: Sec. S1.4. The algorithm uses a last-in-first-out stack to go through the connected parts of the graph, and marks the vertices it encounters as visited. If all vertices have been visited once the stack is empty, the graph is connected.

#### Canonicity test

The most important (and most difficult) part of the enumeration process is the testing of the generated adjacency matrices for canonicity, i.e., assessing whether there is no isomorphic adjacency matrix that is lexicographically larger. The routines to perform this canonicity test are described in Additional file 1: Sec. S1.5.

#### Special treatment of hydrogen atoms

Hydrogen atoms typically represent the most numerous atoms in a molecule. Therefore, it is advantageous to rely on a special treatment for this atom type during the enumeration process [9]. To that end, following the suggestion of Grund [9], the hydrogen atoms are associated to the heavy atom bearing them in a pre-processing step, thereby producing united-atoms with accordingly decreased valences. These are used in the filling algorithm. In this way, the hydrogen atoms are “implicit” during the orderly enumeration, resulting in a significant speed gain. This is described in more detail in Additional file 1: Sec. S1.6.

### Canonical SMILES

The *enu* program enumerates isomers based on lexicographically canonical adjacency matrices. However, for convenience, it reports the generated isomers as canonical SMILES strings [17]. The generation of a unique SMILES representation requires the specification of a canonical atom ordering [20, 21]. Over the past decades, various algorithms have been developed to achieve such a canonicalization [20, 21, 29–32]. While the matrix canonicity criterion of *enu* already leads to a canonical ordering of the atoms, this order is not necessarily suitable to create elegant (i.e., easily readable) SMILES strings. Thus, once a new canonical adjacency matrix is found, a different canonicalization algorithm is used as a post-processing step in *enu* to create the corresponding canonical SMILES strings. It is based on a combination of the schemes proposed by Weininger *et al.* in 1989 [20] and by Schneider *et al.* in 2015 [21]. The applied algorithm is explained in more detail in Additional file 1: Sec. S2. Note that during both the enumeration of the adjacency matrices and the generation of canonical SMILES strings, the hydrogen atoms are treated implicitly.

### Enumeration of stereoisomers

When going from a two- to a three-dimensional representation of a molecule, constitutionally identical molecules can have a different spatial arrangement of their atoms, leading to *stereoisomerism* [24]. In *enu*, the enumeration of the stereoisomers associated with a given constitutional isomer is performed as a postprocessing step to the constitutional-isomer generation. Two kinds of stereoisomerism are considered: (i) chirality is considered for tetravalent atoms that have four singly-bonded neighbours, and (ii) cis/trans stereoisomerism is considered for double bonds connecting two tetravalent atoms that have two singly-bonded neighbors (in addition to the doubly-bonded one) and that are not part of a cycle. Currently, handling of the stereochemistry for possible centers of valence higher than four is not implemented. In addition, double bonds within cycles or cummulene systems are at present not considered in the search of cis/trans stereocenters. Note that the points discussed in the following sections are general observations about stereocenters, and that similar ideas were also developed independently earlier [33].

**Fig. 3:**
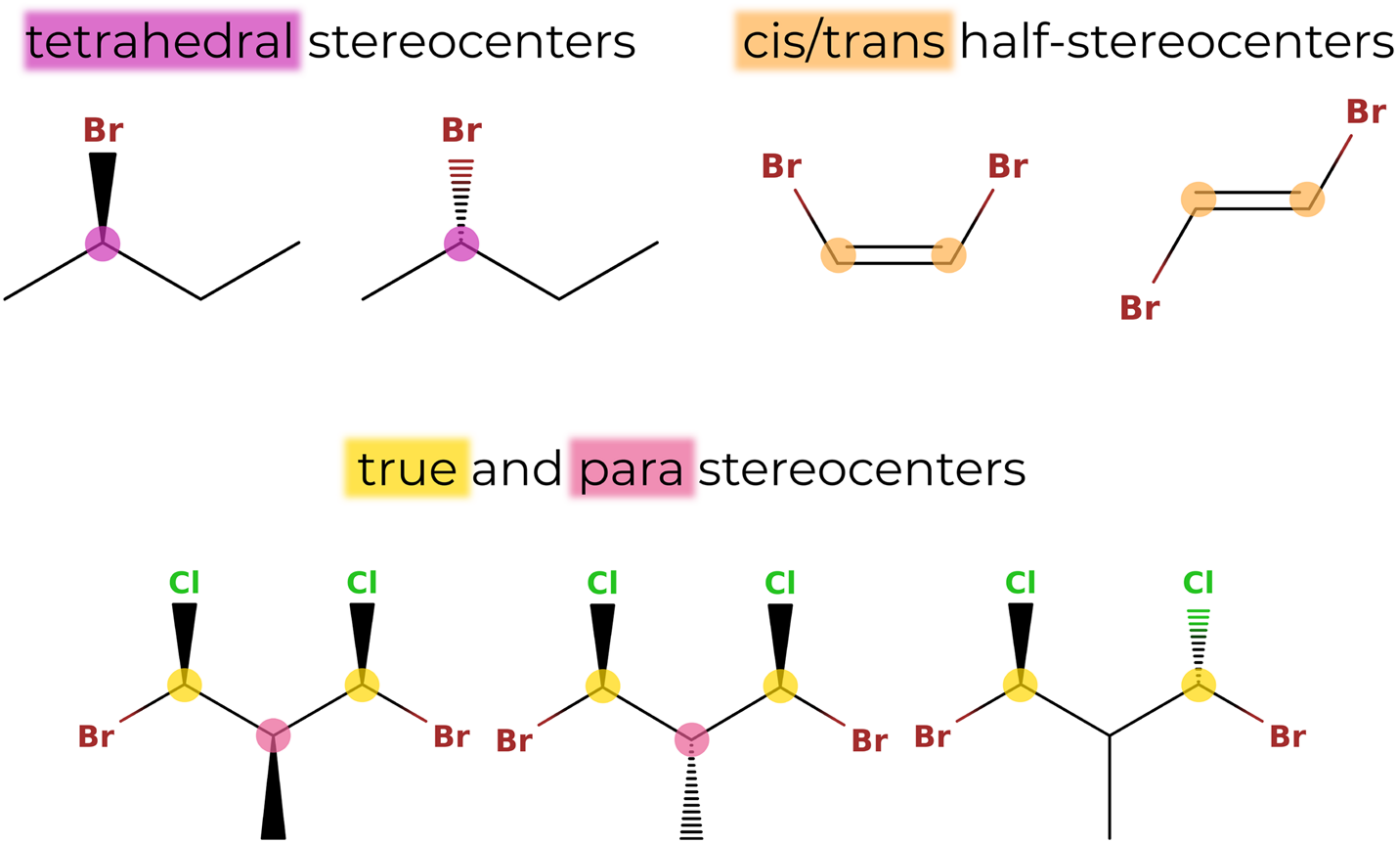
Illustration of the different types of stereocenters. Top: Tetrahedral stereocenters (purple) are bonded to four different substituents, and cis/trans stereocenters (orange) consist of two cis/trans half-stereocents which are bonded by a double bond, and each connected to two different substituents by single bonds. Bottom: Distinction between true (yellow) and para (pink) stereocenters. For a true stereocenter, the substituents differ in their constitution, whereas for a para stereocenter, the substituents only differ in their stereochemistries

In practice, the tetravalent atoms are typically carbon atoms. The neighbors (substituents) can differ either in their constitution, i.e., different atom types or connectivities, or in their spatial arrangements, i.e., different stereo configurations. Stereocenters with neighbors differing in their constitutions are called *true stereocenters*, while stereocenters with neighbors differing only in their stereo configurations are called *para stereocenters* [33]. The considered types of stereocenters (i.e., tetrahedral and cis/trans) as well as the distinction between true and para stereocenters is illustrated in Fig. 3.

Here, stereoisomers will refer to the isomers of a given constitutional isomer corresponding to different spatial arrangements around tetrahedral centers and double bonds. The entire collection of all stereoisomers for all constitutional isomers of a given formula will be referred to as the spatial isomers of the molecule. The *enu* program thus enumerates all constitutional and spatial isomers of a chemical formula.

The procedure to enumerate stereoisomers consists of two steps. In a first step, all unique true stereoisomers are determined. In a second step, the para stereoisomers are generated. This division is necessary because the para stereocenters may be active or not depending on the stereo configuration of the other stereocenters in the molecule. The enumerator uses canonical SMILES strings to represent the enumerated stereoisomers. These strings describe the *local* stereo configuration of the stereocenters in the molecule, which implies that the specified configuration depends on the order in which the atoms appear in the string [17].

#### Finding the true stereoisomers of a molecule

The problem of finding all true stereoisomers of a molecule involves two sequential tasks: (i) identifying the true stereocenters, and (ii) generating all unique true stereoisomers that arise from these stereocenters.

##### Recognizing true stereocenters

**Fig. 4:**
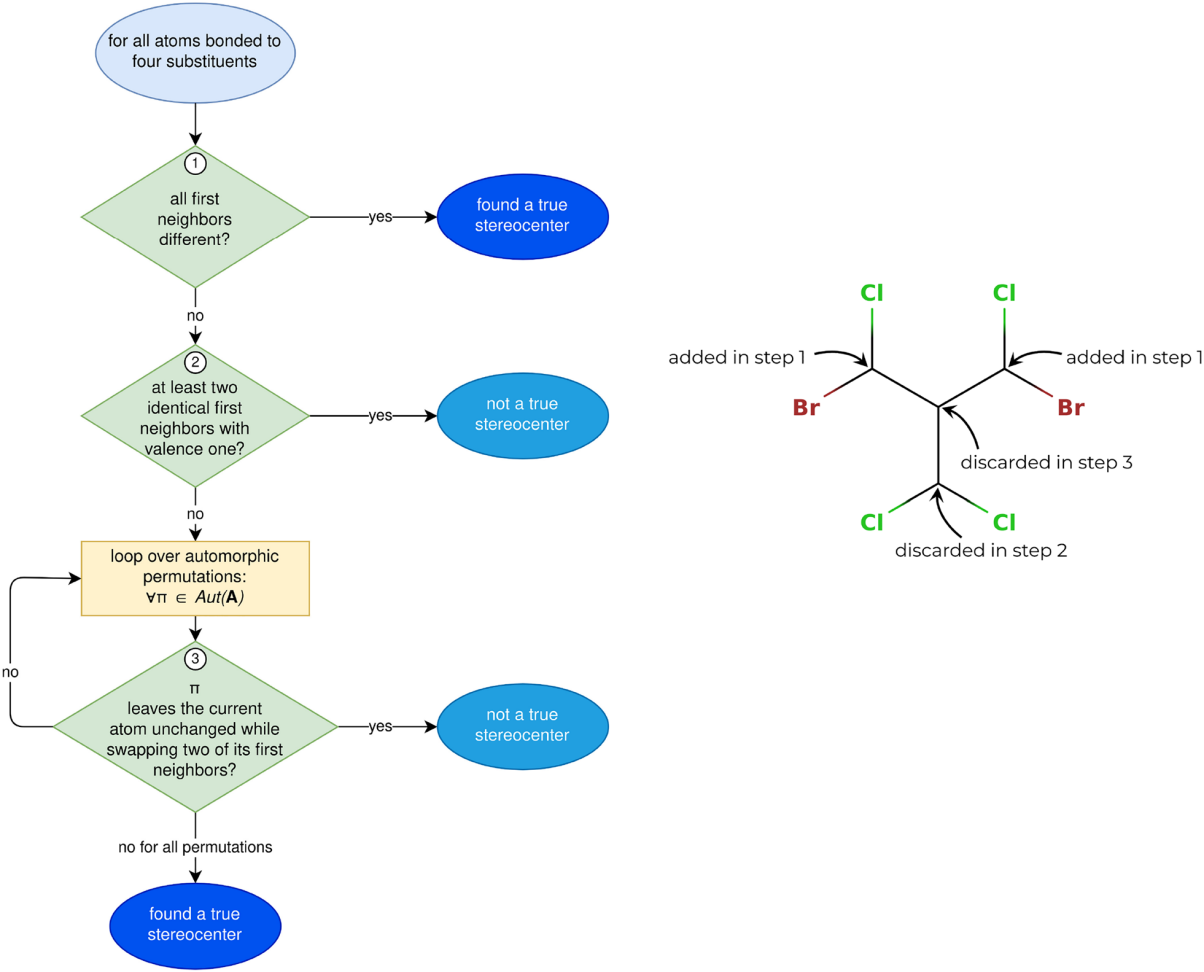
Left: Procedure followed to find all true tetrahedral stereocenters of a molecule. It makes use of the automorphism group $Aut({\textbf{A}})$ of the adjacency matrix which is created during the orderly enumeration process. Right: Illustrative example how the procedure detects the true tetrahedral stereocenters of a molecule. Note that the hydrogen atoms are treated implicitly

 The procedure to detect true *tetrahedral* stereocenters is shown in Fig. 4. The process consists of checking all atoms with four neighbors (or three neighbors and one implicitly connected hydrogen). If the four first-neighbor atoms are all different, a true tetrahedral stereocenter is directly detected. If at least two first neighbors are identical and have valence one, the atom cannot be a true stereocenter. If two or more first neighbors are identical but have a valence larger than one, the algorithm relies on the automorphism group. The automorphism group $Aut({\textbf{A}})$ is generated as a by-product of the canonicity test in the enumeration algorithm (see Additional file 1: Sec. S1.5.5). It contains the atom index permutations which leave the adjacency matrix $\pmb A$ unchanged. If there is at least one permutation $\pi \in Aut({\textbf{A}})$ which leaves the potential stereocenter unchanged (i.e., does not swap it with another atom) but swaps two of its first neighbors, the atom is not a true stereocenter. If such a permutation does not exist, the potential stereocenter is a true tetrahedral stereocenter.

**Fig. 5:**
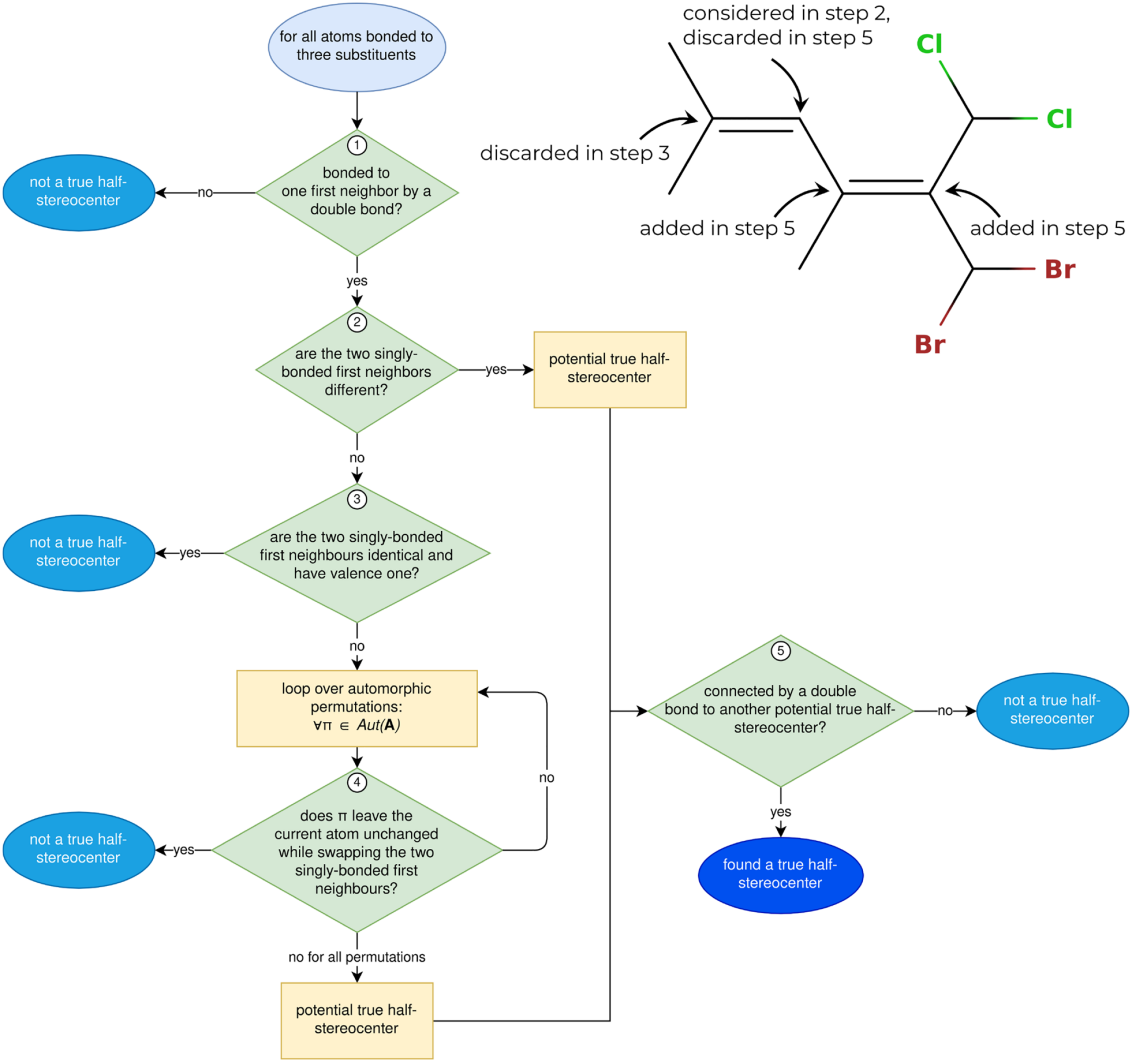
Left: Procedure followed to find all true cis/trans half-stereocenters of a molecule. It makes use of the automorphism group $Aut({\textbf{A}})$ of the adjacency matrix which is created during the orderly enumeration process. Top right: Illustrative example how the procedure detects the true cis/trans stereocenters of a molecule. Note that the hydrogen atoms are treated implicitly

A cis/trans stereocenter is defined as a pair of atoms connected by a double bond. The term (cis/trans) *half-stereocenter* will be used here for these two atoms. The procedure to identify true cis/trans half-stereocenters is analogous to the one for tetrahedral stereocenters. It is outlined in Fig. 5. Here, one considers all the atoms with three neighbors (or two neighbors and one implicitly connected hydrogen atom) that are connected by exactly one double bond to another atom. If the two singly-bonded first-neighbor atoms are identical and have valence one, the considered atom is not a true half-stereocenter. If the two singly-bonded first neighbors are different or if they are identical but there is no permutation $\pi \in Aut({\textbf{A}})$ that leaves the considered atom identical while swapping the two singly-bonded first neighbors, the considered atom is a potential half-stereocenter. All potential half-stereocenters which are connected by a double bond to another potential half-stereocenter are true half-stereocenters.

#### Generating all unique true stereoisomers

**Fig. 6:**
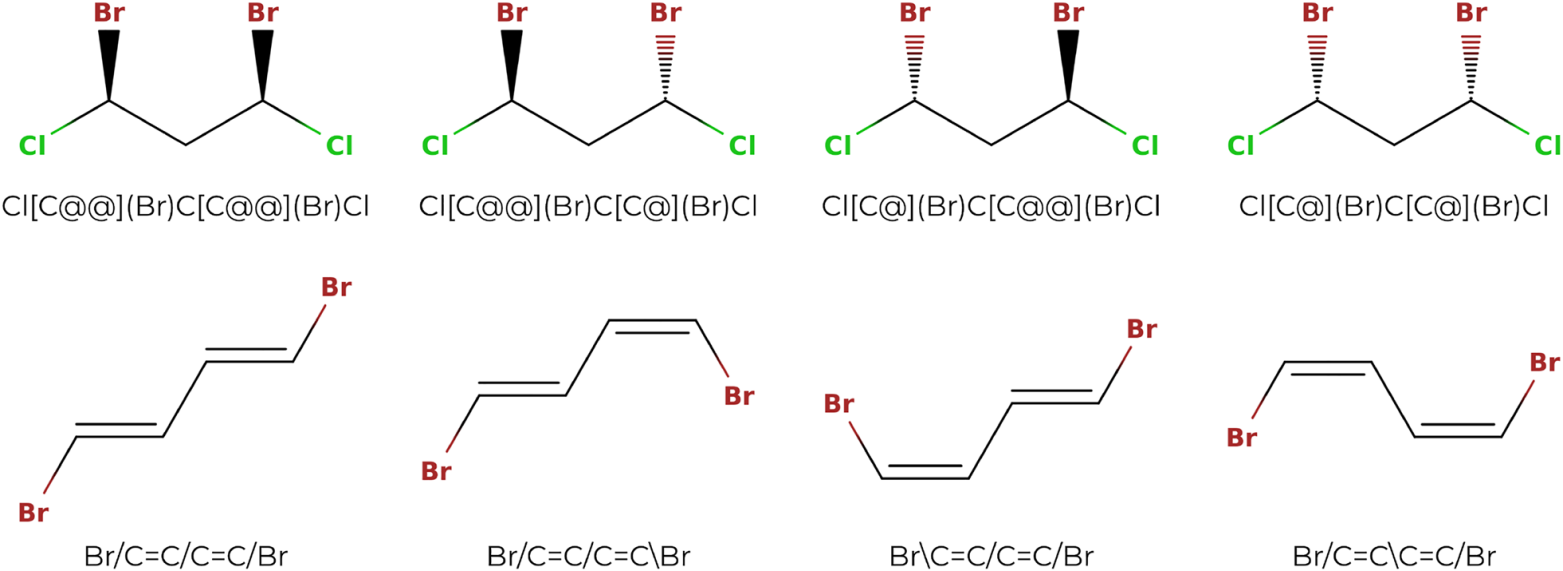
Non-uniqueness of the true stereoisomers generated by binary enumeration. Both of the molecules depicted, ClC(Br)CC(Br)Cl and BrC=CC=CBr, possess two true stereocenters. There are thus $2^2=4$ possible binary configuration vectors. The corresponding stereoisomers are shown for the two molecules. In both cases, however, only three stereoisomers are unique. For the stereoisomers on the top, the stereoisomer with configuration vector [0,0] (leftmost) is identical to that with configuration vector [1,1] (rightmost). For the stereoisomers on the bottom, the stereoisomer with configuration vector [0,1] (left of center) is identical to that with configuration vector [1,0] (right of center)

Once the $n_{tet}$ true tetrahedral stereocenters and the $n_{ct}$ true cis/trans stereocenters of a molecule have been identified, it is straightforward to enumerate the SMILES strings corresponding to the associated $2^{n_{tet} + n_{ct}}$ true stereoisomers. A binary *configuration vector* of size $n_{tet} + n_{ct}$ is used to this purpose. For the tetrahedral centers, a 0 specifies a clockwise direction (encoded by a ‘@@’ in the SMILES string), and a 1 specifies a counter-clockwise direction (encoded by a ‘@’ in the SMILES string). For double bonds, a 0 specifies a trans configuration (encoded either by ‘/’ and ‘/’ or by ‘\’ and ‘\’ in the SMILES string) and a 1 specifies a cis configuration (encoded either by ‘/’ and ‘\’ or by ‘\’ and ‘/’ in the SMILES string). The vector is initially filled with zeros, and binary counting is then used to find all possible configurations of the true stereocenters. However, not all stereoisomers constructed using this approach are unique, as can be seen by considering the examples in Fig. 6.

**Fig. 7:**
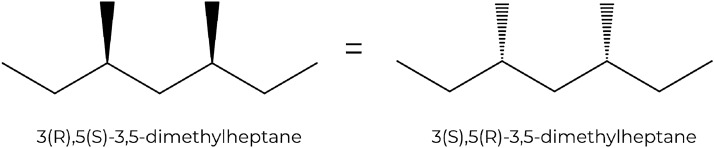
Advantage of absolute chirality notations. This example illustrates that when stereocenters with absolute stereo configurations are swapped, the stereo configuration is simply swapped as well

To make sure that only unique stereoisomers are reported, the automorphism group $Aut(\pmb A)$ is used to filter the results of the binary counting procedure. Here, the convention is used that the smallest equivalent stereochemical configuration vector is reported as the canonical one. For each configuration vector, it has to be determined whether the current stereoisomer is equivalent to one of the previously generated stereoisomers, i.e., one with a lexicographically smaller configuration vector. For all permutations $\pi \in Aut(\pmb A)$, the true stereocenters are checked. By definition, two stereocenters can only be swapped by a permutation of the automorphism group if they are configurationally indistinguishable. If such a swap occurs in a given permutation, the corresponding encoding of 0 or 1 in the configuration vector has to be changed accordingly. If the resulting configuration vector is smaller than the original one, the stereoisomer has already been encountered, and is not counted again. When using a notation that specifies absolute stereo configurations, the step of adapting the encoding in the configuration vector is trivial, as the configurations of the two swapped stereocenters are simply swapped as well (Fig. 7). However, since SMILES strings only specify the stereo configuration locally, the step of adapting the encoding in the configuration vector is not as trivial.

**Fig. 8:**
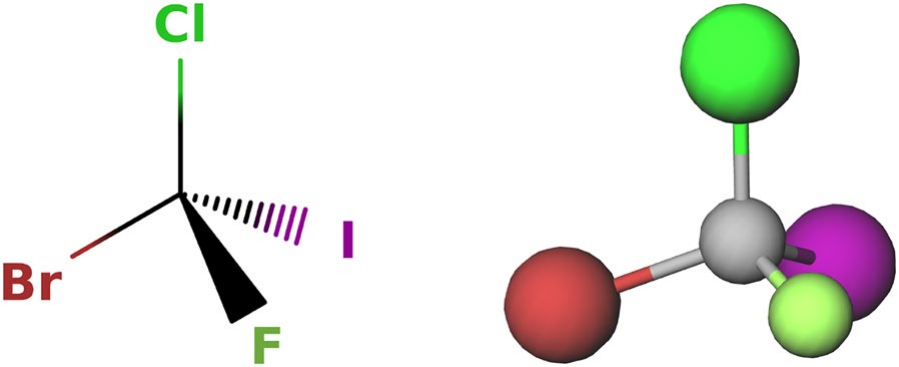
Influence of the order of the atoms in the SMILES strings on the notation for the local stereo configuration. The displayed molecule corresponds to e.g. the SMILES string Br[C@](Cl)(I)F. If the order of the first-neighbor atoms of the central carbon in the string is changed by an odd permutation (odd number of swaps), the stereo configuration notation of the carbon changes from ‘@’ to ‘@@’. For example, if the iodine and the chlorine are swapped, this results in the string Br[C@@](I)(Cl)F. For an even permutation (even number of swaps), the stereo configuration notation stays the same. For example, if the bromine and the chlorine are swapped and then the chlorine and the iodine are swapped, this results in the string I[C@](Br)(Cl)F

**Fig. 9:**
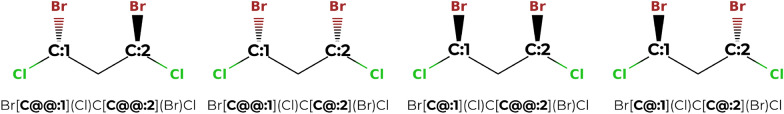
Procedure to detect duplicate stereoisomers. The depicted molecule has two true stereocenters $a_1$ (C:1) and $a_2$ (C:2). Considering the SMILES string Br[C:1](Cl)C[C:2](Br)Cl, there are four possible configuration vectors [0,0], [0,1], [1,0] and [1,1], which correspond to the stereoisomers depicted (from left to right). In a SMILES string, an ‘@’ indicates a counter-clockwise direction and an ‘@@’ a clockwise one (when looking from the atom that comes before the stereocenter in the string towards the stereocenter and then looking at the other three neighbors in the order in which they appear in the string; if there is an implicit hydrogen attached to the stereocenter, it is treated as the first neighbor visited after the stereocenter, or if the stereocenter is the first atom in the SMILES string, as the neighbor visited before the stereocenter [17]). Given the atom ordering retained in the SMILES string, the neighbors of $a_1$ are $\pmb n_1 =$[Br,H,Cl,C], and the neighbors of $a_2$ are $\pmb n_2 =$[C,H,Br,Cl]. To go from the neighbor order of $\pmb n_1$ to the one of $\pmb n_2$, one has to swap Br and C, and then Br and Cl. This corresponds to an even permutation. Thus, if the two stereocenters of Br[C@@:1](Cl)C[C@@:2](Br)Cl are swapped, this results in the string Br[C@@:2](Cl)C[C@@:1](Br)Cl, which is identical to the original one. The same is true for Br[C@:1](Cl)C[C@:2](Br)Cl. On the other hand, if the two stereocenters of Br[C@@:1](Cl)C[C@:2](Br)Cl are swapped, this results in the string Br[C@:2](Cl)C[C@@:1](Br)Cl, which means that the configuration vectors [0,1] and [1,0] correspond to the same stereoisomer. Thus, only the one with the smaller configuration vector [0,1], i.e., Br[C@@:1](Cl)C[C@:2](Br)Cl, will be counted. Finally, the three unique stereoisomers are Br[C@@:1](Cl)C[C@@:2](Br)Cl, Br[C@@:1](Cl)C[C@:2](Br)Cl and Br[C@:1](Cl)C[C@:2](Br)Cl

The local stereo configuration depends on the order in which the first neighbors of a stereocenter appear in the SMILES string, as can be seen in Fig. 8. If a tetrahedral stereocenter is connected to four neighboring atoms (or three atoms and one implicit hydrogen), there are $4!=24$ possible orders (or $3!=6$ in the case of a center with an implicit hydrogen). When a permutation $\pi \in Aut(\pmb A)$ is applied to a stereoisomer, it is possible that a stereocenter is permuted with a different stereocenter and the configuration vector has to be adapted accordingly. For this, the order of the neighboring atoms of the original stereocenter in the SMILES string has to be compared to the corresponding order after applying the permutation. If the stereocenter $a_i$ with neighbors $\pmb n_i = [n_{i,1},n_{i,2},n_{i,3},n_{i,4}]$ (order in the SMILES string) and configuration $c_i \in \lbrace 0,1\rbrace$ is permuted with the stereocenter $a_j$ with neighbors $\pmb n_j = [n_{j,1}, n_{j,2}, n_{j,3}, n_{j,4}]$ and configuration $c_j\in \lbrace 0,1\rbrace$, one determines for each of the neighboring atoms in $\pmb n_i$ the neighboring atoms in $\pmb n_j$ it is permuted with. If the new ordering is an even permutation of the old ordering, the configuration $c_i^{perm}$ of stereocenter $a_i$ in the new configuration vector will be encoded with the old configuration of $a_j$, i.e., $c_i^{perm} = c_j$. Conversely, if the new ordering is an odd permutation, the configuration $c_i^{perm}$ of $a_i$ will be encoded with the opposite of the old configuration of $a_j$, i.e., $c_i^{perm} = \lnot c_j$. Once the permuted configuration vector is generated, it can be decided whether the current stereoisomer is equivalent to a stereoisomer with a smaller configuration vector. If it is the case, the stereoisomer is not counted again. An example how non-unique stereoisomers are detected is shown in Fig. 9.

Unlike a tetrahedral stereocenter, a cis/trans half-stereocenter is only connected to three neighboring atoms (or two atoms and one implicit hydrogen). The configuration of a cis/trans stereocenter is determined by the value of 0 or 1 in the stereochemical configuration vector, as well as by which of the two singly-bonded neighbors are considered for the directionality. Here, the convention is used that the directionality is always specified considering the two singly-bonded neighbors that are encountered first in the SMILES string. If two atoms $a_{i,1}$ and $a_{i,2}$ forming a cis/trans stereocenter $s_i$ with configuration $c_i\in \lbrace 0, 1\rbrace$ (where the singly-bonded neighbors considered for the directionality are the atoms $n_{i,1}$ and $n_{i,2}$) are swapped with two other atoms $a_{j,1}$ and $a_{j,2}$ forming a cis/trans stereocenter $s_j$ with configuration $c_j$ (where the singly-bonded neighbors considered for the directionality are the atoms $n_{j,1}$ and $n_{j,2}$), the new configuration of $s_i$ is determined as follows. If $n_{i,1}$ is swapped with $n_{j,1}$ and $n_{i,2}$ is swapped with $n_{j,2}$, this corresponds to an even permutation, and the configuration of $s_i$ in the new configuration vector will be equal to the configuration of $s_j$, i.e., $c_i^{perm} = c_j$. If $n_{i,1}$ is not swapped with $n_{j,1}$, but $n_{i,2}$ is swapped with $n_{j,2}$ (or vice versa) this means that, due to the ordering of the atoms in the SMILES string, a different pair of singly-bonded first neighbors is considered for the directionality of $s_i$ than for the directionality of $s_j$, and the configuration of $s_i$ in the new vector becomes the opposite of the configuration of $s_j$, i.e., $c_i^{perm} = \lnot c_j$. If neither $n_{i,1}$ is swapped with $n_{j,1}$ nor $n_{i,2}$ with $n_{j,2}$, it means that the opposite pair of first neighbors is considered for the directionality of the configuration of $s_i$ than it was for $s_j$, and the configuration of $s_i$ in the new configuration vector is the same as the configuration of $s_j$ in the old one, i.e., $c_i^{perm} = c_j$.

#### Recognizing para stereocenters

Once the true stereocenters have been found and all unique stereoisomers stemming from these centers have been enumerated, the next step is to complete the list of stereoisomers by adding the para stereoisomers. In the following, the term *potential para stereocenter* is used to denote a center that can possibly be a para stereocenter. Whether this possibility is realized depends on the actual configuration of the true stereocenters (and of the other potential para stereocenters) in the molecule.

An atom is a potential tetrahedral para stereocenter if it was omitted from the list of true stereocenters in the third test of the algorithm in Fig. 4. This corresponds to the situation where at least one permutation $\pi \in Aut(\pmb A)$ leaves the center unchanged while swapping two of its first neighbors. This indicates that the substituents of the center starting with these first neighbors are *constitutionally* identical, but may potentially be *stereochemically* distinct if they encompass true or other para stereocenters in specific configurations.

The same logic applies to potential cis/trans para half-stereocenters. If an atom was discarded in the fourth or fifth test of the algorithm in Fig. 5 it might be a potential cis/trans para half-stereocenter. This is the case if the atom has two identical singly-bonded first neighbors which are swapped by at least one permutation $\pi \in Aut(\pmb A)$, indicating that the two neighboring substituents are *constitutionally* identical, but may be *stereochemically* distinct. The actual para cis/trans stereocenter is defined as a pair of half-stereocenters, so it still has to be checked whether a potential cis/trans para half-stereocenter is connected by a double bond to another potential para cis/trans half-stereocenter.

**Fig. 10:**
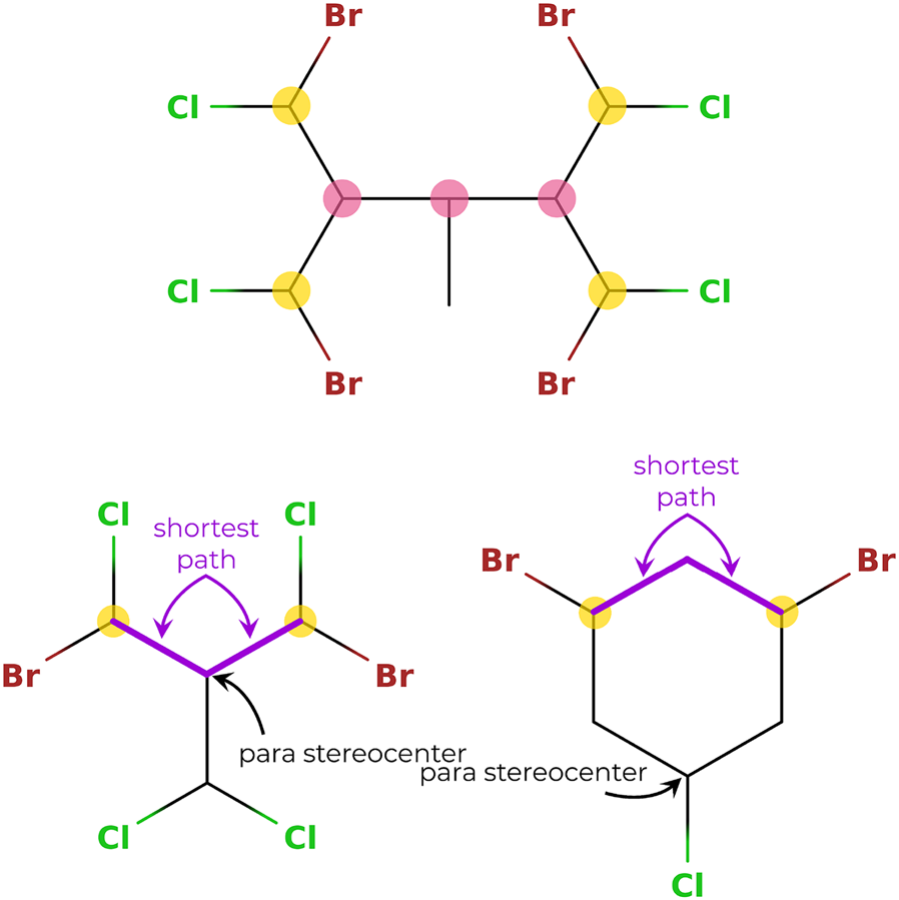
Illustration of para stereocenters. Top: The true stereocenters are marked with a yellow dot and the potential para stereocenters are marked with a pink dot. The hydrogen atoms are not shown explicitly. The two outer potential para stereocenters lie in the middle between the two symmetrical true stereocenters on the left and right, respectively. The inner potential para stereocenter lies in the middle of the two outer potential para stereocenters, as well as of the four combinations of true stereocenters on the two opposite sides of the molecule. Bottom: Illustration of the shortest path and ring criteria for detecting potential para stereocenters

An additional criterion that can be used to reduce the number of potential para stereocenters to be tested is that a para stereocenter lies “in the middle” of at least one pair (or, possibly, multiple pairs) of configurationally symmetrical true stereocenters. Here, symmetrical means that there is at least one permutation $\pi \in Aut(\pmb A)$ which swaps the two true stereocenters. If two symmetrical true stereocenters are not part of a cycle, a simple shortest-path algorithm can be used to determine which atom lies in the middle of the two. If an atom is part of one or more cycles, there is no simple shortest-path algorithm to check all potential paths between two true stereocenters, and this filtering cannot be employed. This is illustrated in Fig. 10. Note also that within a cycle, there can also exist para stereocenters that do not depend on true stereocenters, but only on other para stereocenters, e.g *cis*-1,4-dimethylcyclohexane and *trans*-1,4-dimethylcyclohexane. Such para stereocenters are currently not yet considered in *enu*.

To summarize, the potential para stereocenters that are retained consist of the ones that were discarded as true stereocenters and lie either in a cycle or in the middle of the shortest path between at least one pair of symmetrical true stereocenters. If there are no potential para stereocenters in a molecule, the list of stereoisomers is already complete after considering the true stereoisomers. Otherwise, the list is processed anew to generate the para stereoisomers.

#### Generating all unique para stereoisomers

The process to generate all unique para stereoisomers has to be performed for each true stereoisomer. Whether a potential para stereocenter actually *is* a stereocenter can only be determined once the stereo configuration in a true stereoisomer is specified.

The algorithm to determine all unique para stereoisomers of a molecule goes as follows. For each true stereoisomer, the automorphism group $Aut^{true}(\pmb A)\subseteq Aut(\pmb A)$ is considered, which contains the subset of permutations in the automorphism group that only swap true stereocenters if they have the same absolute stereo configuration. Next, a para configuration vector is created and binary counting is used to find all possible para stereoisomers. For each of these, it first has to be determined which of the potential para stereocenters are actually stereocenters in the current configuration. Then it has to be determined whether this stereoisomer has already been found before, i.e., with a smaller para configuration vector.

In order to determine which potential para stereocenters are actually stereocenters, the automorphism group $Aut^{para}(\pmb A)\subseteq Aut^{true}(\pmb A)$ is considered, which contains the permutations of $Aut^{true}(\pmb A)$ that only swap para stereocenters if they have the same absolute stereo configuration in the current para stereoisomer (i.e., the true stereoisomer with the para stereo configuration specified by the current para configuration vector). All potential para stereocenters for which there exists a permutation $\pi \in Aut^{para}(\pmb A)$ that leaves the current para stereocenter identical but swaps at least two of its immediate neighbors are not stereocenters in the current para stereoisomer. This is indicated by setting the corresponding entry in the para configuration vector to -1 (technically making the vector ternary instead of binary). These steps are repeated as long as at least one para stereocenter is determined to be “not active” in the current configuration.

In order to check whether para stereocenters have the same absolute configuration, the same logic can be used as for true stereocenters. Two symmetrical tetrahedral para stereocenters have the same absolute configuration if (i) the order of the first neighbors of the first stereocenter in the SMILES string is an even permutation of the order of the first neighbors of the second stereocenter in the string and they have the same encoding in the para configuration vector, or (ii) the order of the first neighbors is an odd permutation and they have the opposite encoding in the para configuration vector. Similarly, two symmetrical cis/trans para stereocenters have the same absolute configuration if the two singly-bonded neighbors (and the connected substituents) encountered first in the SMILES string (i) are both the same for the two stereocenters and the stereocenters have the same encoding in the para configuration vector, (ii) are both not the same for the two stereocenters and the stereocenters have the same encoding in the para configuration vector, or (iii) if only one of the singly-bonded neighbors of the first stereocenter is the same as the singly-bonded neighbors of the second stereocenter and the stereocenters have the opposite encoding in the para configuration vector.

**Fig. 11:**
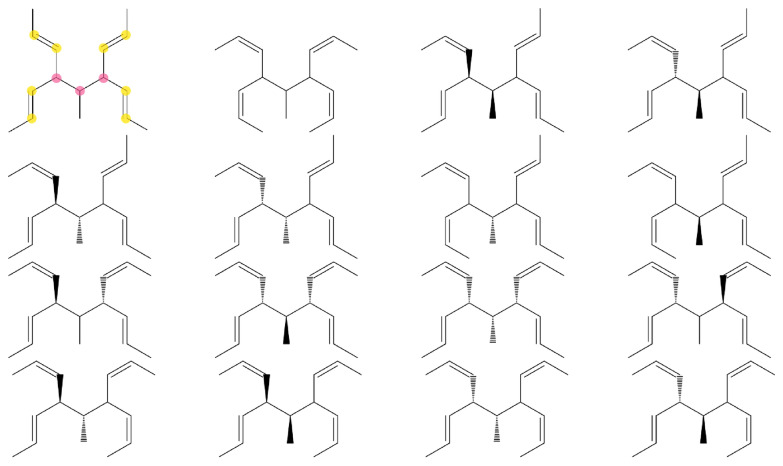
Illustration of para stereoisomers. This relatively small molecule contains four true and three potential para stereocenters, with a total of 16 different stereoisomers. In the molecule on the top left, the true (half-)stereocenters are marked in yellow and the potential para stereocenters are marked in pink. With a total of seven stereocenters there could in principle be $2^7=128$ stereoisomers. However, in many cases, one or more of the potential para stereocenters is not a stereocenter, and multiple stereo configurations actually correspond to the same stereoisomer

The process to eliminate equivalent but smaller para configurations is the same as for true stereocenters. For the lexicographical comparison, the values of -1 in the para configuration vector are treated as 0. Fig. 11 shows the stereoisomers of a small molecule that has four true stereocenters and three potential para stereocenters.

#### Creating SMILES strings for stereoisomers

Once the list of stereoisomers of a constitutional isomer is available as a list of configuration vectors for the true and para stereocenters, this information can be included into the corresponding SMILES strings. For the tetrahedral stereocenters, the encoding is straightforward. In the SMILES string, the element symbol of the stereocenter is enclosed by rectangular brackets, and either ‘@@’ or ‘@’ is added if the corresponding element in the configuration vector has the value 0 or 1, respectively. Additionally, if the stereocenter is connected to an implicit hydrogen atom, a ‘H’ is added before the closing rectangular bracket.

**Fig. 12:**
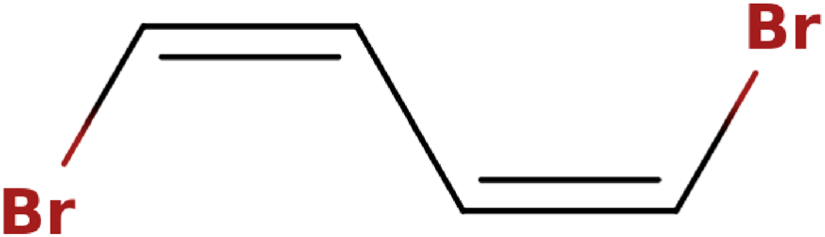
Illustration of a potential issue with generating cis/trans SMILES strings. The depicted molecule, described by the SMILES string BrC=CC=CBr, contains two true cis/trans stereocenters. The configuration vector is [1, 1], where 1 corresponds to a cis configuration. If the configuration is included in the SMILES string by adding a ‘/’ before the first visited half-stereocenters, and adding a ‘\’ before the first visited singly-bonded neighbors of the two second half-stereocenters, the SMILES string would become Br/C=C\/C=C\Br, which is not valid. Instead, the cis/trans half stereocenters are processed in the order in which they appear in the string. For each of the half-stereocenters, it is then checked whether the corresponding first visited half-stereocenter already possesses an encoding. In this example, the string would be built as Br/C=C, then before the singly-bonded neighbor of the second carbon atom, the encoding ‘\’ is added, leading to Br/C=C\ (i.e., cis). Then, the string continues to be processed until the next cis/trans half-stereocenter is encountered, leading to Br/C=C\C=C. Since the singly-bonded neighbor of the third carbon atom is already assigned the encoding ‘\’, the corresponding encoding before the singly-bonded neighbor of the fourth carbon atom is chosen accordingly, leading to the final string Br/C=C\C=C/Br (i.e., cis and cis)

The handling of cis/trans stereocenters is slightly more complicated. In the general case, one simply adds ‘/’ before the half-stereocenter that is visited first in the SMILES string, and ‘/’ or ‘\’ before the first visited singly-bonded neighbor of the second half-stereocenter if the corresponding element in the configuration vector has the value 0 or 1, respectively. However, the situation is more complicated if a half-stereocenter is also the singly-bonded neighbor of another half-stereocenter (Fig. 12). A simple way to handle this situation is to go through the cis/trans stereocenters in the order in which the half-stereocenters are visited in the SMILES string, checking if the first half-stereocenter already contains an encoding, and then adapting the encoding of the first visited singly-bonded neighbor of the second half-stereocenter accordingly (i.e., cis or trans).

In the XML output, the number of tetrahedral and cis/trans stereocenters is reported for each stereoisomer. Additionally, for molecules with at least one tetrahedral stereocenter, the enantiomer of each stereoisomer is reported (if one exists).

### Implementation details

The functionalities described in the previous sections are implemented in a C++ program called the *isomer enumerator*, in short *enu*. The current version of the program can be found on GitHub at https://github.com/csms-ethz/CombiFF. This repository also contains input and output files, as well as other programs related to the CombiFF scheme [15, 16]. Running enu -help will print an overview of the input options to the standard output. A user manual for *enu* is also provided in https://github.com/csms-ethz/CombiFF/blob/main/doc/enu.pdf.

The following sections provide an overview over some of the functionalities implemented into the enumerator.

#### Specifying a molecular formula

There is some flexibility to define how many times an element type should occur in the molecular formula. For each element type, this number can be given as a single integer (e.g. H5), as a list of integers (e.g. H[0,2,4,5]), or as a range (e.g. H[0-5]).

#### Implicit hydrogen atoms

As described previously, hydrogen atoms are treated implicitly (i.e., distributed among the other atom types before the enumeration algorithm starts). In order to specify the types of molecules of interest more distinctly, the implicit hydrogen atoms can also be specified directly in the chemical formula. For example, {CH1}1{CH2}2{OH1}3 will be translated to the formula $\hbox {C}_{3}\hbox {H}_{8}\hbox {O}_{3}$ with the restriction that it includes one carbon atom bonded to exactly one hydrogen atom, two carbon atoms bonded each to exactly two hydrogen atoms, and three oxygen atoms bonded to exactly one hydrogen atom. This will generate the two constitutional isomers OCC(O)CO and OCCC(O)O (no stereoisomers), whereas there exist 36 constitutional and spatial isomers for the unrestricted formula $\hbox {C}_{3}\hbox {H}_{8}\hbox {O}_{3}$.

#### Filtering for properties

Currently, the user may restrict the following molecular properties: The maximum bond order, the number of unsaturations (summing one for double bonds and cycles, and two for triple bonds), the total number of bonds (each bond counted once, irrespective if single or multiple), the number of single bonds, the number of double bonds, the number of triple bonds, the number of quadruple bonds, as well as the number of cycles in the molecule. These restrictions can be set either as an integer, as a list of integers, or as a range of integers.

The filtering for the number of unsaturations is performed before the enumeration starts, by calculating the number of unsaturations for a molecular formula using Eq. S16 (see Additional file 1). The filtering for the other properties is done whenever a new isomer is found. If the restrictions are not met, the isomer is not reported.

For example, enumerating all straight-chain alkane isomers $\hbox {C}_{\hbox {n}}\hbox {H}_{2n+2}$ from $\hbox {C}_{1}\hbox {H}_{4}$ to $\hbox {C}_{20}\hbox {H}_{42}$ could be achieved with the formula specification C[1-20]H[4-42] and the restriction that the number of unsaturations should be zero.

#### Aromaticity

Currently, there is only a very basic implementation to recognize aromatic rings of size six using a substructure search for alternating single and double bonds. This procedure is able to recognize structures like benzene and pyridine, which is sufficient to eliminate duplicate isomers where the ordering of the single and double bonds is different. However, this functionality is still very limited. In the future, it will be extended to recognize aromatic rings during the enumeration procedure. In the meantime, it is possible to post-process the *enu* output using a suitable cheminformatics library such as the *RDKit* [34] to recognize aromaticity for more complicated cases. Thanks to the convenient XML output format and the SMILES notation, such a post-processing is easy to implement.

#### Visualization

**Fig. 13:**
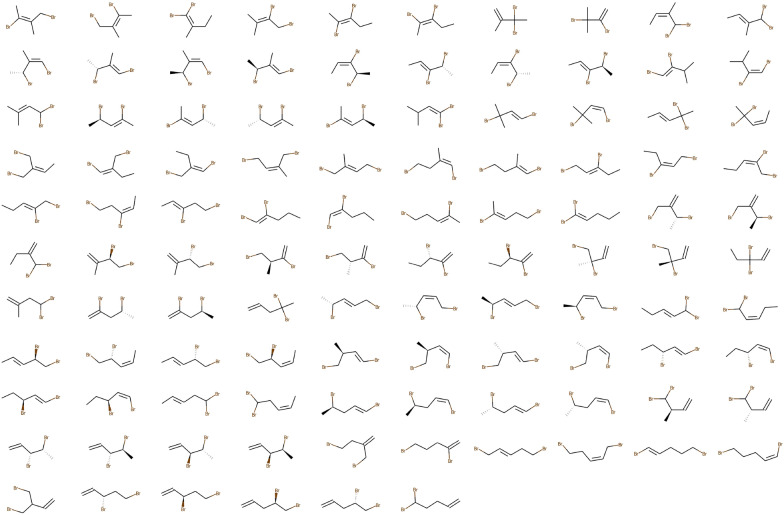
Visualization of the 106 acyclic constitutional and spatial isomers of $\hbox {C}_{5}\hbox {H}_{8}\hbox {Br}_{2}$. The molecules were enumerated with *enu* and the depiction was generated with the *RDKit* [34] cheminformatics library

To visualize the output of *enu*, a small Python script is provided in the GitHub repository (Fig. 13). It uses the Python3 [35] *xml.etree.ElementTree* module to parse the XML list of constitutional and spatial (if present) isomers, the Python library *pdfrw* [36] to concatenate PDFs, as well as the *RDKit* [34] cheminformatics library to create the visualizations (for an example, see Fig. 13).

#### Family definitions

The most straightforward way to use *enu* is *via* the command line by specifying a chemical formula (potentially including atoms with implicit hydrogens and filtering criteria, as described in the previous sections). However, one can also define a so-called *family* which offers more flexibility, i.e., the use of element aliases, of a filtering for substructures, and of pseudoatoms. A brief overview is given in the next paragraphs.

##### Element aliases

 In order to provide more flexibility in the definition of the chemical formulas for which the isomers are to be enumerated, it is possible to define so called *element aliases*. An element alias has a name and contains a set of element types. For example, an element alias for the halogen element types could be called “Hal” and contain the four types “Br”, “Cl”, “F” and “I”. When two or more of the same element alias occur in a chemical formula, AND, XOR, and OR logic can be used to specify if they should be of the same element type, of a different element type, or whether both is allowed. For example, the notation Hal3 (AND) specifies that the halogen atoms have to be the same type (*e.g.*, Br3). ${\hat{\,}}$ Hal1${\hat{\,}}$ Hal2 (XOR) specifies that the first halogen atom has to be of a different type than the two other halogen atoms (e.g., Br1Cl2). Finally, Hal1Hal1Hal1 (OR) specifies that any combination of three halogen atoms is allowed (e.g*.*, Br1Cl1F1, Br3, or Br1Cl2). The formula to enumerate all straight-chain haloalkanes with ten carbon atoms and two halogen atoms of the same type can then be expressed as C10H20Hal2 and is equivalent to enumerating the four chemical formulae $\hbox {C}_{10}\hbox {H}_{20}\hbox {Br}_2$, $\hbox {C}_{10}\hbox {H}_{20}\hbox {Cl}_{2}$, $\hbox {C}_{10}\hbox {H}_{20}\hbox {F}_2$ and $\hbox {C}_{10}\hbox {H}_{20}\hbox {I}_2$.

##### Filtering for substructures

 It is also possible to filter for the occurrence of substructures. The number of occurrences can be specified by a single integer, a list of numbers, or a range of numbers. By setting the occurrence to zero, one may also prevent the occurrence of a substructure. The implemented substructure search algorithm is the Ullmann algorithm [37]. As the enumerator is aimed for relatively small molecules, the performance of the Ullmann algorithm is not a bottleneck for the overall runtime. If this became an issue in the future, it could be replaced by a more modern algorithm such as VF2 [38, 39].

Here, a substructure is defined by a name, a list of atoms, and an adjacency matrix stack (i.e., the upper triangle of an adjacency matrix, written row-wise as a one-dimensional vector). Each of the atoms can be either an element type, an element type with a number of implicit hydrogens, an element alias, or a wildcard. If there is more than one element alias of the same type, it is also possible to use the AND, XOR and OR logic to specify if they should be of the same element types, of a different element type, or whether both are allowed. When element aliases are used and multiple occurrences of the substructure are requested, it is also possible to specify how the element types should occur across substructures, also using AND, XOR and OR logic.

When multiple substructures are required, the implemented convention is that there can be a maximum overlap of one atom between the matched substructures. For example, in the molecule CCCC, the substructure CC is found three times, but the substructure CCC is only found once.

##### Pseudoatoms

 Applying substructure matching in the form of a post-processing step as described in the previous section can become very inefficient if a chemical formula is given with many potential isomers, where only a small subset of them contain the desired substructure(s).

Consider, for example, the formula C[2-10]H[2-18]O4, where the number of unsaturations in the molecule is set to two. In total, there exist more than 560 million constitutional and spatial isomers. However, if one requires exactly two occurrences of the substructure COC(=O)H, only 1484 of these isomers contain the desired substructures. The enumeration of these isomers takes about 12 minutes on a laptop with an i7-8565U CPU. A major part of the computation time is thus wasted on constitutional isomers which are not reported. Note that there is no time wasted on enumerating redundant stereoisomers, as the stereoisomers are only generated for the constitutional isomers that are compatible with the given restrictions.

The number of redundant constitutional isomers can be reduced by using implicit hydrogens specifying the formula C[0-8]{CH1}2H[2-18]{OH0}4, such that isomers containing e.g. an oxygen–hydrogen bond are not generated during the enumeration. With this more specific formula, the enumeration time reduces to about 1 min. However, for the larger example C[4-10]H[4-16]O6, where the number of unsaturations in the molecule is set to three and it is required that there are exactly three occurrences of the substructure COC(=O)H, even with this trick of using the formula C[0-7]{CH1}3H[4-16]{OH0}6, the enumeration of the the 1328 constitutional and spatial isomers takes about 50 minutes.

To solve this problem in a more general fashion, so-called *pseudoatoms* are introduced. A pseudoatom is a molecular substructure which only contains one atom that is not fully bonded, i.e., can be connected to the atoms of the rest of the molecule. A pseudoatom is defined by a name, a list of atoms and an adjacency matrix stack. The pseudoatom behaves like a normal atom during the enumeration process. The valence of the pseudoatom corresponds to the valence of the not-fully-bonded atom minus the number of bonds that this atom forms with the other atoms within the pseudoatom. Whenever a new isomer is found, the pseudoatom is explicited in terms of its atom content, i.e., the adjacency matrix is extended and canonicalized, the SMILES string is generated, and the stereoisomers are listed (if requested). Using a pseudoatom OC(=O)H, it takes less than a second to enumerate the 1328 constitutional and spatial isomers of C[1-7]H[4-16]’OC(=O)H’3 with three occurrences of the substructure COC(=O)H, i.e., much less than the above 50 min.

A caveat of this approach is that the canonicity test does not recognize if a pseudoatom can also be constructed using the atoms available in the rest of the molecule. When this is possible, there will be duplicate isomers in the enumeration list. However, based on to the canonical SMILES strings these duplicates can easily be removed by the user in a post-processing step. Thanks to the convenient XML output format and the SMILES notation, such a post-processing is easy to implement if required.

#### Output

The enumerated isomers are written to an output file following an XML format. The corresponding *Document Type Definition* (DTD) can be found at https://github.com/csms-ethz/CombiFF/blob/main/use/output_files/isomer_enumeration.dtd. In addition to the generated output file, the program prints the current molecular formula as well as the current number of isomers during the enumeration process to the standard output (updated after every 100th detected (stereo)isomer). It is also possible to count the number of (stereo)isomers without generating the list of SMILES strings as an XML output file, by using the argument -count_only.

## Illustrative results

**Fig. 14:**
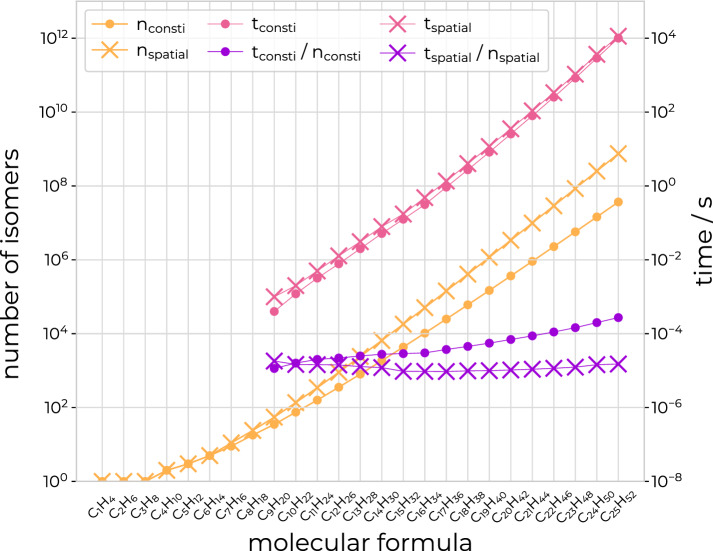
Illustration of the performance of the enumerator. The graph exemplifies the performance of the isomer enumerator in the context of the straight-chain alkanes $\hbox {C}_{n}\hbox {H}_{2n+2}$ from $\hbox {C}_{1}\hbox {H}_{4}$ to $\hbox {C}_{25}\hbox {H}_{52}$. It shows the number of alkane (stereo)isomers as a function of the number of carbon atoms (yellow), the wall-clock time to count these (stereo)isomers (pink), and the wall-clock time spent per (stereo)isomer for the counting (purple). The left vertical axis shows the number of (stereo)isomers and the right vertical axis shows the elapsed wall-clock time. All calculations were performed on AMD EPYC 7763 CPUs of the ETH Zürich Euler cluster [40] and averaged over five runs. Note that values with $t<0.001$ s are not displayed. The numerical values are provided in Table 1

The performance of the isomer enumerator is illustrated in the context of straight-chain alkanes from $\hbox {C}_{1}\hbox {H}_{4}$ to $\hbox {C}_{24}\hbox {H}_{50}$. All time measurements are taken from runs performed on AMD EPYC 7763 CPUs of the ETH Zürich Euler cluster (2.6 GHz nominal, 3.3 GHz peak frequency, 256 GB of DDR4 memory clocked at 3200 MHz) [40] and averaged over five runs. Table 1 lists the run times and number of enumerated constitutional and spatial isomers for the alkanes. Figure 14 shows the number of constitutional and spatial alkane isomers as a function of the number of carbon atoms, the time to enumerate these isomers, and the time spent per constitutional/spatial isomer during the enumeration process depending on the number of carbon atoms. The number of existing constitutional/spatial isomers, and thus also the wall-clock time, increases exponentially upon increasing the number of carbon atoms. The time spent per constitutional isomer also increases exponentially, though with a much smaller slope. The time spent per spatial isomer remains essentially constant up to at least 25 carbon atoms.

**Table 1:** Run times of the isomer enumerator for alkane isomers

Molecule	$n_\text {consti}$	$n_\text {spatial}$	$t_\text {consti}$ [s]		$t_\text {spatial}$ [s]	
			No output	SMILES	No output	SMILES
$\hbox {C}_{1}\hbox {H}_{4}$	1	1	< 0.01	< 0.01	< 0.01	< 0.01
$\hbox {C}_{2}\hbox {H}_{6}$	1	1	< 0.01	0.01	< 0.01	0.01
$\hbox {C}_{3}\hbox {H}_{8}$	1	1	< 0.01	< 0.01	< 0.01	< 0.01
$\hbox {C}_{4}\hbox {H}_{10}$	2	2	< 0.01	< 0.01	< 0.01	< 0.01
$\hbox {C}_{5}\hbox {H}_{12}$	3	3	< 0.01	0.01	< 0.01	< 0.01
$\hbox {C}_{6}\hbox {H}_{14}$	5	5	< 0.01	< 0.01	< 0.01	< 0.01
$\hbox {C}_{7}\hbox {H}_{16}$	9	11	< 0.01	< 0.01	< 0.01	< 0.01
$\hbox {C}_{8}\hbox {H}_{18}$	18	24	< 0.01	< 0.01	< 0.01	< 0.01
$\hbox {C}_{9}\hbox {H}_{20}$	35	55	< 0.01	< 0.01	< 0.01	< 0.01
$\hbox {C}_{10}\hbox {H}_{22}$	75	136	< 0.01	< 0.01	< 0.01	< 0.01
$\hbox {C}_{11}\hbox {H}_{24}$	159	345	< 0.01	0.01	< 0.01	0.01
$\hbox {C}_{12}\hbox {H}_{26}$	355	900	< 0.01	0.01	0.01	0.02
$\hbox {C}_{13}\hbox {H}_{28}$	802	2412	0.02	0.03	0.03	0.05
$\hbox {C}_{14}\hbox {H}_{30}$	1858	6563	0.05	0.07	0.08	0.11
$\hbox {C}_{15}\hbox {H}_{32}$	4347	18127	0.13	0.18	0.17	0.31
$\hbox {C}_{16}\hbox {H}_{34}$	10359	50699	0.31	0.40	0.48	0.70
$\hbox {C}_{17}\hbox {H}_{36}$	24894	143255	0.93	1.16	1.35	2.48
$\hbox {C}_{18}\hbox {H}_{38}$	60523	408429	2.74	3.17	4.02	9.86
$\hbox {C}_{19}\hbox {H}_{40}$	148284	1173770	8.22	9.37	11.70	28.48
$\hbox {C}_{20}\hbox {H}_{42}$	366319	3396844	25.55	33.87	35.33	99.87
$\hbox {C}_{21}\hbox {H}_{v44}$	910726	9892302	79.41	104.05	107.88	280.27
$\hbox {C}_{22}\hbox {H}_{46}$	2278658	28972080	253.57	273.27	334.91	869.78
$\hbox {C}_{23}\hbox {H}_{48}$	5731580	85289390	834.42	927.93	1060.18	1338.33
$\hbox {C}_{24}\hbox {H}_{50}$	14490245	252260276	2895.93	2997.59	3649.41	4545.92
$\hbox {C}_{25}\hbox {H}_{52}$	36797588	749329719	10040.19	10455.26	11309.32	13179.80

The table shows the number $n_\text {consti}$ of constitutional isomers and the number $n_\text {spatial}$ of spatial isomers of the straight-chain alkanes from $\hbox {C}_{1}\hbox {H}_{4}$ to $\hbox {C}_{24}\hbox {H}_{50}$, as well as the wall-clock time spent on their enumeration. The time $t_\text {consti}$ is the wall-clock time in seconds needed to enumerate the constitutional isomers, and the time $t_\text {spatial}$ is the wall-clock time in seconds needed to enumerate the spatial isomers. The elapsed wall-clock time is reported both for the case when no output is generated as well as the case when the SMILES strings of the isomers are reported. All calculations were performed on AMD EPYC 7763 CPUs of the ETH Zürich Euler cluster [40] and averaged over five runs. The results are illustrated graphically in Fig. 14

**Fig. 15:**
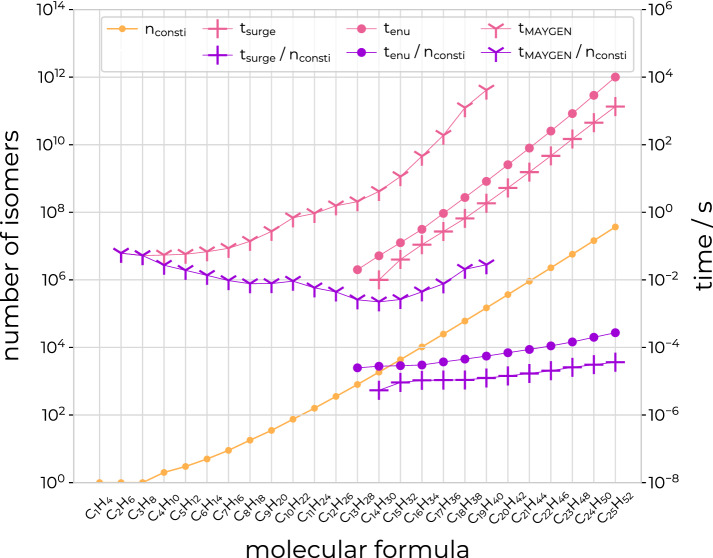
Comparison of the performance of the enumerators. The graph compares the performance of *enu* (dots) to the programs *surge* (pluses) and *MAYGEN* (tri-downs) in the context of the straight-chain alkanes $\hbox {C}_{n}\hbox {H}_{2n+2}$ from $\hbox {C}_{1}\hbox {H}_{4}$ to $\hbox {C}_{25}\hbox {H}_{52}$. The graph shows the number of constitutional alkane isomers as a function of the number of carbon atoms (yellow), the wall-clock time to count these isomers (pink), and the wall-clock time spent per isomer for the counting (purple). The left vertical axis shows the number of isomers and the right vertical axis shows the elapsed wall-clock time. All calculations were performed on AMD EPYC 7763 CPUs of the ETH Zürich Euler cluster [40] and averaged over five runs. Note that the *MAYGEN* calculations were stopped after exceeding $10^4$ s and values with $t<0.01$ s are not displayed. The numerical values are provided in Table 2

**Fig. 16:**
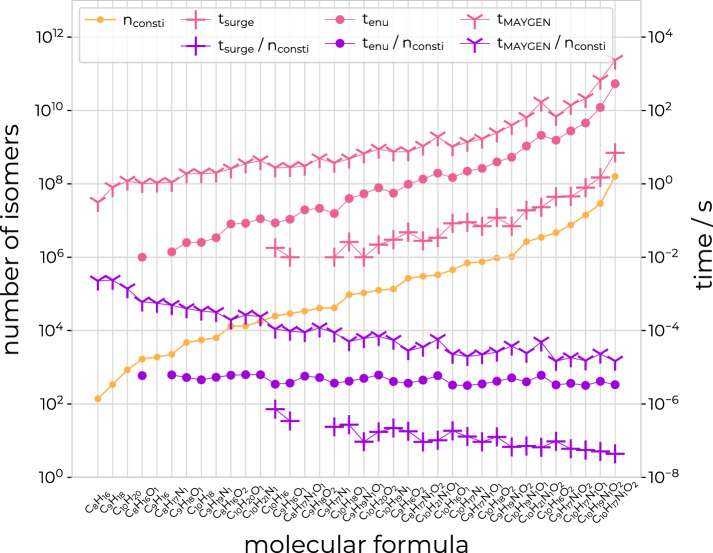
Comparison of the performance of the enumerators. The graph compares the performance of *enu* (dots) to the programs *surge* (pluses) and *MAYGEN* (tri-downs) in the context of molecular formulas with 8–10 carbon atoms, 0–1 nitrogen atoms, 0–2 oxygen atoms, and 16–21 hydrogen atoms, and with the number of unsaturations set to either zero or one. The molecular formulas are sorted by the number of detected isomers. The graph shows the number of constitutional alkane isomers depending on the number of carbon atoms (yellow), the wall-clock time to count these isomers (pink), and the wall-clock time spent per isomer for the counting (purple). The left vertical axis shows the number of isomers and the right vertical axis shows the elapsed wall-clock time. All calculations were performed on AMD EPYC 7763 CPUs of the ETH Zürich Euler cluster [40] and averaged over five runs. Note that values with $t<0.01$ s are not displayed. The numerical values are provided in Table 3

The performance of *enu* is compared to the two recently published open-source structure generators *MAYGEN* [12] (version 1.8) and *surge* [13] (version 1.0). The program *MAYGEN* is based on the same principles of orderly enumeration as *enu* and is written in Java. The program *surge* is written in C and uses the *nauty* package [41] to generate molecular graphs in an efficient three-step process [13]. Both programs count or enumerate the constitutional isomers of a given molecular formula. The elapsed wall-clock time for the enumeration using the three programs is reported for the straight-chain alkanes from $\hbox {C}_{1}\hbox {H}_{4}$ to $\hbox {C}_{25}\hbox {H}_{52}$ (Table 2; Fig. 15) as well as for molecular formulas with 8-10 carbon atoms, 0–1 nitrogen atoms, 0–2 oxygen atoms, and 16–21 hydrogen atoms, and with the number of unsaturations set to either zero or one (Table 3; Fig. 16). All calculations were performed on AMD EPYC 7763 CPUs of the ETH Zürich Euler cluster [40], using one core per calculation, and averaged over five runs. The elapsed wall-clock time is reported both for the case when no output is generated as well as the case when the SMILES strings of the isomers are reported. For all of the tested molecular formulas, *surge* is the fastest of the three programs, followed by *enu*, and then by *MAYGEN*. For the molecular formulas of Table 3, the maximum used memory to count the isomers was 6 MB (*surge*), 11 MB (*enu*), or 1154 MB (*MAYGEN*), respectively. The average used memory was 6 MB (*surge*), 10 MB (*enu*), or 640 MB (*MAYGEN*), respectively. For the tested molecular formulas, the three programs reported the same number of isomers.

**Table 2:** Comparison of run times for alkane isomers

Molecule	$n_\text {consti}$	$t_\text {surge}$ [s]		$t_\text {enu}$ [s]		$t_\text {MAYGEN}$ [s]	
		No output	SMILES	No output	SMILES	No output	SMILES
$\hbox {C}_{1}\hbox {H}_{4}$	1	< 0.01	< 0.01	< 0.01	< 0.01	< 0.01	0.10
$\hbox {C}_{2}\hbox {H}_{6}$	1	< 0.01	< 0.01	< 0.01	0.01	0.06	0.14
$\hbox {C}_{3}\hbox {H}_{8}$	1	< 0.01	< 0.01	< 0.01	< 0.01	0.05	0.13
$\hbox {C}_{4}\hbox {H}_{10}$	2	< 0.01	< 0.01	< 0.01	< 0.01	0.05	0.13
$\hbox {C}_{5}\hbox {H}_{12}$	3	< 0.01	< 0.01	< 0.01	0.01	0.06	0.16
$\hbox {C}_{6}\hbox {H}_{14}$	5	< 0.01	< 0.01	< 0.01	< 0.01	0.07	0.17
$\hbox {C}_{7}\hbox {H}_{16}$	9	< 0.01	< 0.01	< 0.01	< 0.01	0.09	0.20
$\hbox {C}_{8}\hbox {H}_{18}$	18	< 0.01	< 0.01	< 0.01	< 0.01	0.14	0.29
$\hbox {C}_{9}\hbox {H}_{20}$	35	< 0.01	< 0.01	< 0.01	< 0.01	0.27	0.52
$\hbox {C}_{10}\hbox {H}_{22}$	75	<0.01	< 0.01	< 0.01	< 0.01	0.69	0.96
$\hbox {C}_{11}\hbox {H}_{24}$	159	< 0.01	< 0.01	< 0.01	0.01	0.92	1.44
$\hbox {C}_{12}\hbox {H}_{26}$	355	< 0.01	< 0.01	< 0.01	0.01	1.56	2.64
$\hbox {C}_{13}\hbox {H}_{28}$	802	< 0.01	< 0.01	0.02	0.03	2.06	4.31
$\hbox {C}_{14}\hbox {H}_{30}$	1858	0.01	0.02	0.05	0.07	4.15	6.91
$\hbox {C}_{15}\hbox {H}_{32}$	4347	0.04	0.04	0.13	0.18	11.41	20.04
$\hbox {C}_{16}\hbox {H}_{34}$	10359	0.11	0.12	0.31	0.40	45.79	74.30
$\hbox {C}_{17}\hbox {H}_{36}$	24894	0.27	0.29	0.93	1.16	190.40	279.11
$\hbox {C}_{18}\hbox {H}_{38}$	60523	0.66	0.71	2.74	3.17	1247.30	1637.19
$\hbox {C}_{19}\hbox {H}_{40}$	148284	1.85	1.98	8.22	9.37	4146.88	6124.98
$\hbox {C}_{20}\hbox {H}_{42}$	366319	5.27	5.63	25.55	33.87	–	–
$\hbox {C}_{21}\hbox {H}_{44}$	910726	15.45	16.39	79.41	104.05	–	–
$\hbox {C}_{22}\hbox {H}_{46}$	2278658	46.73	49.14	253.57	273.27	–	–
$\hbox {C}_{23}\hbox {H}_{48}$	5731580	147.63	154.90	834.42	927.93	–	–
$\hbox {C}_{24}\hbox {H}_{50}$	14490245	449.20	465.13	2895.93	2997.59	–	–
$\hbox {C}_{25}\hbox {H}_{52}$	36797588	1347.30	1388.39	10040.19	10455.26	–	–

The table shows the number $n_\text {consti}$ of constitutional isomers of the straight-chain alkanes from $\hbox {C}_{1}\hbox {H}_{4}$ to $\hbox {C}_{25}\hbox {H}_{52}$, as well as the wall-clock time spent on their enumeration using the three programs *surge* ($t_\text {surge}$), *enu* ($t_\text {enu}$), and *MAYGEN* ($t_\text {MAYGEN}$). The elapsed wall-clock time is reported both for the case when no output is generated as well as the case when the SMILES strings of the isomers are reported. All calculations were performed on AMD EPYC 7763 CPUs of the ETH Zürich Euler cluster [40] and averaged over five runs. Note that the *MAYGEN* calculations were stopped after exceeding $10^4$ s. The results are illustrated graphically in Fig. 15

**Table 3:** Comparison of *enu* with *surge* and *MAYGEN*. The table shows the number $n_\text {consti}$ of constitutional isomers for molecular formulas with 8–10 carbon atoms, 0–1 nitrogen atoms, 0–2 atoms, and 16–21 hydrogen atoms, and with the number of unsaturations set to either zero or one. The molecular formulas are sorted by the number of detected isomers. Further, the wall-clock time spent on the enumeration is reported when using the three programs *surge* ($t_\text {surge}$), *enu* ($t_\text {enu}$), and *MAYGEN* ($t_\text {MAYGEN}$). The elapsed wall-clock time is reported both for the case when no output is generated as well as the case when the SMILES strings of the isomers are reported. All calculations were performed on AMD EPYC 7763 CPUs of the ETH Zürich Euler cluster [40] and averaged over five runs. The results are illustrated graphically in Fig. 16

Molecule	$n_\text {consti}$	$t_\text {surge}$ [s]		$t_\text {enu}$ [s]		$t_\text {MAYGEN}$ [s]	
		No output	SMILES	No output	SMILES	No output	SMILES
$\hbox {C}_{8}\hbox {H}_{16}$	139	< 0.01	< 0.01	< 0.01	0.01	0.31	0.69
$\hbox {C}_{9}\hbox {H}_{18}$	338	< 0.01	< 0.01	< 0.01	0.02	0.80	1.33
$\hbox {C}_{10}\hbox {H}_{20}$	852	< 0.01	< 0.01	< 0.01	0.02	1.18	2.41
$\hbox {C}_{8}\hbox {H}_{16}\hbox {O}_{1}$	1684	< 0.01	< 0.01	0.01	0.03	1.02	2.95
$\hbox {C}_{9}\hbox {H}_{16}$	1902	< 0.01	< 0.01	< 0.01	0.03	1.06	2.77
$\hbox {C}_{8}\hbox {H}_{17}\hbox {N}_{1}$	2258	< 0.01	< 0.01	0.01	0.04	1.10	3.57
$\hbox {C}_{9}\hbox {H}_{18}\hbox {O}_{1}$	4745	< 0.01	< 0.01	0.03	0.06	1.89	4.46
$\hbox {C}_{10}\hbox {H}_{18}$	5568	<0.01	< 0.01	0.03	0.07	1.90	4.37
$\hbox {C}_{9}\hbox {H}_{19}\hbox {N}_{1}$	6355	< 0.01	< 0.01	0.03	0.08	1.99	5.94
$\hbox {C}_{8}\hbox {H}_{16}\hbox {O}_{2}$	13190	< 0.01	< 0.01	0.08	0.19	2.55	5.10
$\hbox {C}_{10}\hbox {H}_{20}\hbox {O}_{1}$	13372	< 0.01	< 0.01	0.08	0.17	3.55	6.40
$\hbox {C}_{10}\hbox {H}_{21}\hbox {N}_{1}$	17884	<0.01	0.01	0.11	0.21	4.28	7.46
$\hbox {C}_{10}\hbox {H}_{16}$	24938	0.02	0.02	0.09	0.25	2.74	6.59
$\hbox {C}_{9}\hbox {H}_{16}\hbox {O}_{1}$	29172	0.01	0.01	0.11	0.27	2.81	6.24
$\hbox {C}_{8}\hbox {H}_{17}\hbox {N}_{1}\hbox {O}_{1}$	34156	<0.01	0.01	0.20	0.39	3.04	7.40
$\hbox {C}_{9}\hbox {H}_{18}\hbox {O}_{2}$	41039	<0.01	0.01	0.22	0.43	4.86	8.90
$\hbox {C}_{9}\hbox {H}_{17}\hbox {N}_{1}$	41989	0.01	0.02	0.16	0.39	3.67	8.70
$\hbox {C}_{10}\hbox {H}_{18}\hbox {O}_{1}$	95312	0.03	0.04	0.40	0.95	4.79	11.69
$\hbox {C}_{9}\hbox {H}_{19}\hbox {N}_{1}\hbox {O}_{1}$	106849	0.01	0.02	0.53	1.07	6.57	13.00
$\hbox {C}_{10}\hbox {H}_{20}\hbox {O}_{2}$	126750	0.02	0.04	0.78	1.50	8.79	17.31
$\hbox {C}_{10}\hbox {H}_{19}\hbox {N}_{1}$	136086	0.03	0.05	0.56	1.36	7.41	14.16
$\hbox {C}_{9}\hbox {H}_{16}\hbox {O}_{2}$	265782	0.05	0.09	0.98	2.44	7.54	20.32
$\hbox {C}_{8}\hbox {H}_{17}\hbox {N}_{1}\hbox {O}_{2}$	301851	0.03	0.07	1.35	2.83	10.66	24.76
$\hbox {C}_{10}\hbox {H}_{21}\hbox {N}_{1}\hbox {O}_{1}$	330823	0.03	0.07	1.96	3.79	19.10	38.29
$\hbox {C}_{10}\hbox {H}_{16}\hbox {O}_{1}$	452458	0.08	0.15	1.49	4.14	10.23	27.87
$\hbox {C}_{10}\hbox {H}_{17}\hbox {N}_{1}$	693577	0.09	0.20	2.22	6.27	13.64	38.70
$\hbox {C}_{9}\hbox {H}_{17}\hbox {N}_{1}\hbox {O}_{1}$	749685	0.07	0.18	2.64	6.75	16.56	42.38
$\hbox {C}_{10}\hbox {H}_{18}\hbox {O}_{2}$	949780	0.12	0.25	3.96	9.66	24.65	61.51
$\hbox {C}_{9}\hbox {H}_{19}\hbox {N}_{1}\hbox {O}_{2}$	1034078	0.07	0.19	5.32	10.80	39.16	86.92
$\hbox {C}_{10}\hbox {H}_{19}\hbox {N}_{1}\hbox {O}_{1}$	2665421	0.19	0.52	10.72	26.44	64.31	167.69
$\hbox {C}_{10}\hbox {H}_{21}\hbox {N}_{1}\hbox {O}_{2}$	3479760	0.23	0.66	21.09	41.56	169.72	354.46
$\hbox {C}_{10}\hbox {H}_{16}\hbox {O}_{2}$	4676149	0.45	1.08	15.51	43.71	68.55	216.48
$\hbox {C}_{9}\hbox {H}_{17}\hbox {N}_{1}\hbox {O}_{2}$	7558025	0.45	1.28	27.60	70.72	138.02	389.79
$\hbox {C}_{10}\hbox {H}_{17}\hbox {N}_{1}\hbox {O}_{1}$	14167376	0.79	2.56	45.44	132.80	213.13	659.56
$\hbox {C}_{10}\hbox {H}_{19}\hbox {N}_{1}\hbox {O}_{2}$	29182992	1.49	5.12	121.01	301.69	672.95	1788.84
$\hbox {C}_{10}\hbox {H}_{17}\hbox {N}_{1}\hbox {O}_{2}$	159815906	7.00	27.23	536.38	1561.10	2346.42	7434.74

## Conclusions

The goal of this article was to document the algorithms and implementation underlying the program *enu* for the enumeration of the constitutional isomers and stereoisomers of a molecular formula. Although the motivation underlying the development of this program was its integration into the CombiFF workflow [15, 16], *enu* is a stand-alone, freely-downloadable and open-source program, which can be used for any other purpose in cheminformatics. An integration into other workflows can be easily achieved thanks to the convenient XML format and the reporting of isomers via canonical SMILES strings.

The illustrative example of the alkane isomers shows that the computational cost grows exponentially with the number of carbon atoms, just as the number of isomers. However, while the time spent per constitutional isomer also tends to increase exponentially, the time spent per spatial isomer stays essentially constant up to at least 25 carbon atoms. Comparison with the two open-source structure generators *MAYGEN* and *surge* show that *enu* is slower than *surge* and slightly faster than *MAYGEN*. Compared to *MAYGEN* and *surge*, *enu* has the advantage of providing the possibility to enumerate the spatial isomers in addition to the constitutional ones.

Further development of the *enu* program will include: (i) handling the stereochemical properties (chirality, double bonds) within cyclic systems; (ii) identifying aromaticity more comprehensively in the SMILES string generation; (iii) simplifying the input mechanism for atoms with variable valences (e.g. sulfur or phosphorous); (iii) extending the special treatment of hydrogen atoms to all singly-connected entities (halogens, pseudoatoms, methyl groups) for computational efficiency.

## Availability and requirements

- Project name: isomer enumerator.
- Project home page: https://github.com/csms-ethz/CombiFF
- Operating system: Linux (should also work on other platforms, but has not been tested).
- Programming language: C++.
- Other requirements: C++11 compiler, CMake (version 2.8.12 or higher).
- License: BSD-3.

## Supplementary Information

**Additional file 1**. Further details on the theory of the described isomer enumerator.

## Data Availability

The complete source code is available at https://github.com/csms-ethz/CombiFF, version at time of writing: v1.0-beta (https://github.com/csms-ethz/CombiFF/releases/tag/v1.0-beta).
