# Supplementary material for: Development of an open-source software for isomer enumeration
Source: J Cheminform. 2023 Jan 22;15:10. doi: 10.1186/s13321-022-00677-6 (PMC9867865; doi:10.1186/s13321-022-00677-6)
Supplement: Supplementary file 1 — Additional file 1. Further details on the theory of the described isomer enumerator. [file 13321_2022_677_MOESM1_ESM.pdf]

SUPPORTING INFORMATION
Development of an Open-Source Software for Isomer Enumeration

Salomé R. Rieder,*[a] Marina P. Oliveira,[a] Sereina Riniker,[a] and Philippe H. Hünenberger[a]

[a] *Laboratory of Physical Chemistry, ETH Zürich, Vladimir-Prelog-Weg 2, 8093 Zürich, Switzerland. E-mail: phil@ethz.ch*

Contents

S1 Enumeration **S2**
S1.1 Molecular Graphs S2
S1.2 Molecular Graph Isomorphism S3
S1.3 Filling Algorithm to Enumerate Adjacency Matrices S5
S1.4 Connectivity Test S8
S1.5 Canonicity Test S10
S1.5.1 Permutation Trees S10
S1.5.2 Representation Systems S10
S1.5.3 Naïve Canonicity Test S11
S1.5.4 Blockwise Canonicity Test S12
S1.5.5 Pruning the Permutation Tree: Stabilizers S13
S1.5.6 Semi-Canonicity S14
S1.5.7 Index Jumps S15
S1.6 Special Treatment of Hydrogen Atoms S15
S1.7 Comparison to Brute-Force Approach S19

S2 SMILES canonicalization **S22**

S1 Enumeration

S1.1 Molecular Graphs

The definitions and notations adopted here are largely inspired from those of Ref. [1]. A molecular graph is a connected labeled multigraph in which the vertices represent atoms and the edges account for the (single or multiple) covalent bonds between the atoms [2]. An example is shown in Main Text Figure 1. The *label vector* $\boldsymbol{\alpha} = (\alpha_0, \alpha_1, \ldots, \alpha_K)$ of a molecular graph describing a molecule with $K+1$ atom types contains a list of these types (element symbols), each of them appearing only once. The *valence vector* $\boldsymbol{\delta} = (\delta_0, \delta_1, \ldots, \delta_K)$ describes the fixed valences of the atom types in $\boldsymbol{\alpha}$. For elements capable of presenting different valences (*e.g.*, S and P), the corresponding atom type can be split across different valences by associating it to different entries in the label and valence vectors. The *partition vector* $\boldsymbol{\lambda} = (\lambda_0, \lambda_1, \ldots, \lambda_K)$ of the graph is defined such that λ_k corresponds to the number of occurrences of the element type α_k in the molecule [1]. Consequently, the total number N of atoms is given by

$$N = \sum_{k=0}^{K} \lambda_k \,. \tag{S1}$$

The combination of a label vector $\boldsymbol{\alpha}$ and a partition vector $\boldsymbol{\lambda}$ corresponds to a molecular formula, which can be written as ${\alpha_0}_{\lambda_0} {\alpha_1}_{\lambda_1} \cdots {\alpha_K}_{\lambda_K}$. Such a combination can be used to create an *atom vector*

$$\boldsymbol{a} = (a_0, a_1, \ldots, a_{N-1}) = (\underbrace{\alpha_0, \ldots, \alpha_0}_{\lambda_0 \times}, \underbrace{\alpha_1, \ldots, \alpha_1}_{\lambda_1 \times}, \cdots, \underbrace{\alpha_K, \ldots, \alpha_K}_{\lambda_K \times}) \,. \tag{S2}$$

The corresponding *degree vector*

$$\boldsymbol{d} = (d_0, d_1, \ldots, d_{N-1}) = (\underbrace{\delta_0, \ldots, \delta_0}_{\lambda_0 \times}, \underbrace{\delta_1, \ldots, \delta_1}_{\lambda_1 \times}, \cdots, \underbrace{\delta_K, \ldots, \delta_K}_{\lambda_K \times}) \tag{S3}$$

contains the valence of each atom in the molecule, *i.e.* the number of covalent bonds it can form. The atoms in the atom vector are numbered with consecutive indices $i = 0, 1, 2, \ldots, N-1$. The indices of the λ_k atoms with the same label α_k are collected in the so-called *partition* p_k. There are $K+1$ such partitions

$$\begin{aligned}
p_0 &= \{0, 1, \ldots, \lambda_0 - 1\} \\
p_1 &= \{\lambda_0, \lambda_0 + 1, \ldots, \lambda_0 + \lambda_1 - 1\} \\
&\ldots \\
p_k &= \left\{ \sum_{l=0}^{k-1} \lambda_l, \sum_{l=0}^{k-1} \lambda_l + 1, \ldots, \sum_{l=0}^{k} \lambda_l - 1 \right\} \\
&\ldots \\
p_K &= \left\{ \sum_{l=0}^{K-1} \lambda_l, \sum_{l=0}^{K-1} \lambda_l + 1, \ldots, N - 1 \right\} ,
\end{aligned} \tag{S4}$$

and the number of elements in partition p_k is equal to λ_k.

A molecular graph can be described by the combination of a label vector $\boldsymbol{\alpha}$, a valence vector $\boldsymbol{\delta}$, a partition vector $\boldsymbol{\lambda}$, and an *adjacency matrix* $\boldsymbol{A} \in \mathbb{N}_0^{+N \times N}$. A matrix element $A_{i,j}$ of $\boldsymbol{A}$ describes the order of the bond possibly connecting the atom at position i to the atom at position j in the atom vector (or is

set to zero in the absence of a bond). To be compatible with the degree vector, the adjacency matrix must satisfy

$$\sum_{j=0}^{N-1} A_{i,j} = \sum_{j=0}^{N-1} A_{j,i} = d_i \forall i\,. \tag{S5}$$

S1.2 Molecular Graph Isomorphism

For a given choice of $\boldsymbol{\alpha}$, $\boldsymbol{\delta}$, and $\boldsymbol{\lambda}$ (*i.e.*, of a molecular formula and of atom-type valences), the specification of an adjacency matrix $\boldsymbol{A}$ (*i.e.*, of a covalent connectivity between the atoms) defines a unique labeled molecular graph. However, since the atoms of a common type in a molecule are physically indistinguishable, two labeled graphs that are directly related by a permutation in the labels of these atoms actually describe the same molecule (merely with a different atom numbering). In other words, for a given choice of $\boldsymbol{\alpha}$, $\boldsymbol{\delta}$, and $\boldsymbol{\lambda}$, the same molecule can generally be represented by many different adjacency matrices $\boldsymbol{A}$. This observation is fundamentally important to the problem of isomer enumeration and is known as (molecular) graph isomorphism.

A *permutation* π is a linear ordering of the elements of a set, *i.e.*, any list of all the elements of the set in which each element appears exactly once [3]. The set of all permutations of a set with N elements is called the *symmetric group* S_N and contains $N!$ elements [3]. Here, the relevant permutations operate on the set $\{0,1,\ldots,N-1\}$ of indices of the atom vector. Permutations can be formulated as a chain of successive transpositions (swaps), denoted by corresponding index tuples (pairs), *i.e.* as

$$(0,j_0)(1,j_1)(2,j_2)\cdots(N-2,j_{N-2})(N-1,N-1)\,, \tag{S6}$$

where a tuple (i,j_i) indicates that the atom at index i is to be swapped with the atom at index j_i in the atom vector. The transpositions are perfomed in sequence from left to right, and the restriction $j_i \geq i$ is imposed for each tuple (i,j_i). In addition, the two indices in a tuple must be contained in the same index partition p_k (*i.e.* they must swap atoms of the same type). Tuples of the form (i,i) leave the position of index i identical and can thus (but do not have to) be left out of the chain of index tuples.

For a molecular graph with a partition vector $\boldsymbol{\lambda} = (\lambda_0,\lambda_1,\ldots,\lambda_K)$, there are $\lambda_k!$ ways to arrange the indices of the atoms in the *k-th* partition. Consequently, there are $\lambda_0!\cdot\lambda_1!\cdot\ldots\cdot\lambda_K!$ possibilities to arrange all the indices of the atom vector within their respective index partitions.[4] The set of all these permutations is noted by $S_{\boldsymbol{\lambda}}$ and is a subset of the symmetric group S_N. As a result, these resulting permutations leave the vectors $\boldsymbol{a}$ and $\boldsymbol{d}$ unchanged. These permutations affect the adjacency matrix $\boldsymbol{A}$. Applying an index transposition (i,j_i) to a matrix corresponds to swapping rows i and j_i as well as columns i and j_i. The new adjacency matrix may differ from the original one, but it still describes the same molecule, just with a different ordering of the atom indices.

Considering two labeled molecular graphs defined by $(\boldsymbol{\alpha},\boldsymbol{\delta},\boldsymbol{\lambda},\boldsymbol{A})$ and by $(\boldsymbol{\alpha}',\boldsymbol{\delta}',\boldsymbol{\lambda}',\boldsymbol{A}')$, the graphs are called *compatible* if and only if

$$\boldsymbol{\alpha} = \boldsymbol{\alpha}' \text{ and } \boldsymbol{\delta} = \boldsymbol{\delta}' \text{ and } \boldsymbol{\lambda} = \boldsymbol{\lambda}'\,, \tag{S7}$$

i.e., they correspond to the same molecular formula. Two graphs are called *isomorphic* if and only if they are compatible and [1]

$$\exists \pi \in S_{\boldsymbol{\lambda}} : \boldsymbol{A}\pi = \boldsymbol{A}'\,. \tag{S8}$$

In this case, one also says that the corresponding adjacency matrices are isomorphic (noted $\boldsymbol{A}' \sim \boldsymbol{A}$). Two isomorphic graphs are equivalent representations of the same molecules, as illustrated in Figure S1. Note

that identity ($\boldsymbol{A}' = \boldsymbol{A}$) is a special case of isomorphism. Finally, two graphs are called *isomeric* if they are compatible but not isomorphic. Two isomeric graphs describe molecules with the same chemical formulas but that are structurally different [5].

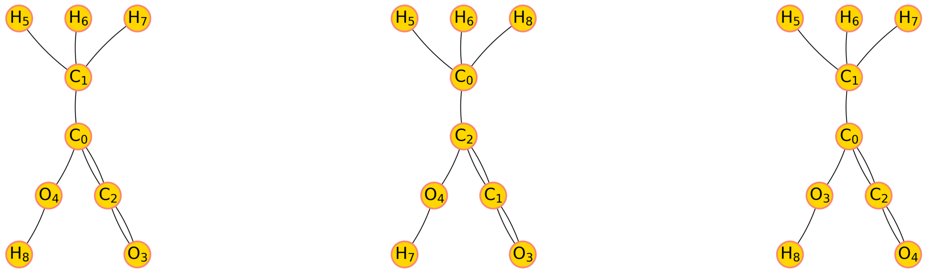

$$\mathbf{A}_1 = \begin{array}{c} \\ \end{array}\begin{array}{ccccccccc} C_0 & C_1 & C_2 & O_3 & O_4 & H_5 & H_6 & H_7 & H_8 \end{array}$$

$$\mathbf{A}_1 = \begin{pmatrix} 0 & 1 & 2 & 0 & 1 & 0 & 0 & 0 & 0 \\ 1 & 0 & 0 & 0 & 0 & 1 & 1 & 1 & 0 \\ 2 & 0 & 0 & 2 & 0 & 0 & 0 & 0 & 0 \\ 0 & 0 & 2 & 0 & 0 & 0 & 0 & 0 & 0 \\ 1 & 0 & 0 & 0 & 0 & 0 & 0 & 0 & 1 \\ 0 & 1 & 0 & 0 & 0 & 0 & 0 & 0 & 0 \\ 0 & 1 & 0 & 0 & 0 & 0 & 0 & 0 & 0 \\ 0 & 1 & 0 & 0 & 0 & 0 & 0 & 0 & 0 \\ 0 & 0 & 0 & 0 & 1 & 0 & 0 & 0 & 0 \end{pmatrix} \begin{matrix} C_0 \\ C_1 \\ C_2 \\ O_3 \\ O_4 \\ H_5 \\ H_6 \\ H_7 \\ H_8 \end{matrix}$$

$$\mathbf{A}_2 = \begin{pmatrix} 0 & 0 & 1 & 0 & 0 & 1 & 1 & 0 & 1 \\ 0 & 0 & 2 & 2 & 0 & 0 & 0 & 0 & 0 \\ 1 & 2 & 0 & 0 & 1 & 0 & 0 & 0 & 0 \\ 0 & 2 & 0 & 0 & 0 & 0 & 0 & 0 & 0 \\ 0 & 0 & 1 & 0 & 0 & 0 & 0 & 1 & 0 \\ 1 & 0 & 0 & 0 & 0 & 0 & 0 & 0 & 0 \\ 1 & 0 & 0 & 0 & 0 & 0 & 0 & 0 & 0 \\ 0 & 0 & 0 & 0 & 1 & 0 & 0 & 0 & 0 \\ 1 & 0 & 0 & 0 & 0 & 0 & 0 & 0 & 0 \end{pmatrix} \begin{matrix} C_0 \\ C_1 \\ C_2 \\ O_3 \\ O_4 \\ H_5 \\ H_6 \\ H_7 \\ H_8 \end{matrix}$$

$$\mathbf{A}_3 = \begin{pmatrix} 0 & 1 & 2 & 1 & 0 & 0 & 0 & 0 & 0 \\ 1 & 0 & 0 & 0 & 0 & 1 & 1 & 1 & 0 \\ 2 & 0 & 0 & 0 & 2 & 0 & 0 & 0 & 0 \\ 1 & 0 & 0 & 0 & 0 & 0 & 0 & 0 & 1 \\ 0 & 0 & 2 & 0 & 0 & 0 & 0 & 0 & 0 \\ 0 & 1 & 0 & 0 & 0 & 0 & 0 & 0 & 0 \\ 0 & 1 & 0 & 0 & 0 & 0 & 0 & 0 & 0 \\ 0 & 1 & 0 & 0 & 0 & 0 & 0 & 0 & 0 \\ 0 & 0 & 0 & 1 & 0 & 0 & 0 & 0 & 0 \end{pmatrix} \begin{matrix} C_0 \\ C_1 \\ C_2 \\ O_3 \\ O_4 \\ H_5 \\ H_6 \\ H_7 \\ H_8 \end{matrix}$$

Figure S1: Illustration of molecular graph isomorphism. Three isomorphic molecular graphs and their corresponding adjacency matrix, representing an isomer of $C_3O_2H_4$ including atom indices.

In order to have a unique representation of the molecular topology in the form of a labeled multigraph, a *lexicographical ordering* can be used as canonicity criterion for the adjacency matrix. An adjacency matrix $\boldsymbol{A}$ is lexicographically larger than an adjacency matrix $\boldsymbol{A}'$ (noted $\boldsymbol{A} > \boldsymbol{A}'$) provided that [1]

$$\exists i_0, j_0 : \left(A_{i,j} = A'_{i,j} \;\wedge\; \left(A_{i_0,j_0} > A'_{i_0,j_0} \right) \;\forall (i,j) < (i_0, j_0) \right) , \tag{S9}$$

with the definition [1]

$$(i,j) < (k,l) \;\Leftrightarrow\; (i < k) \vee (i = k \wedge j < l) \,. \tag{S10}$$

In plain words, when the two matrices are read row-by-row from the top left to the bottom right, the first difference encountered determines the lexicographical ordering.

The canonical adjacency matrix of a molecular graph is then defined as the lexicographically largest among all possible adjacency matrices, which in turn defines a canonical labeling of the atoms in the molecular graph. Thus, for a given choice of $\boldsymbol{\alpha}$, $\boldsymbol{\delta}$, and $\boldsymbol{\lambda}$, an adjacency matrix $\boldsymbol{A}$ is canonical if and only if

$$\nexists \pi \in S_{\boldsymbol{\lambda}} : \boldsymbol{A}\pi > \boldsymbol{A} \,. \tag{S11}$$

For a unique representation of molecules, the canonicity criterion for $\boldsymbol{A}$ must be accompanied by a canonicity criterion for the ordering of the atom types in the vector $\boldsymbol{\alpha}$. The ordering adopted in the *enu* program is as follows. The atom types are sorted from highest to lowest valence δ_k. If multiple atom types have the same valence, they are sorted according to the size of their partitions (*i.e.* the number λ_k of occurrences of the atom in the molecule) in increasing order. If multiple isovalent atom types have the same partition sizes, they are sorted by their atomic number in increasing order. For example, for $C_1O_1N_1Cl_1Br_3F_1$, one would order C, N, O, F, Cl, Br. This specific choice of ordering in terms of valence and occurrence can lead to considerable performance increases during the enumeration process (see Sec. S1.5.4).

With these definitions, a molecule can be uniquely represented as a labeled molecular graph with a canonical adjacency matrix. An example is provided for the molecule depicted in Figure S2 with

$$\boldsymbol{\alpha} = (\mathrm{C}, \mathrm{H}) \tag{S12}$$
$$\boldsymbol{\delta} = (4, 1) \tag{S13}$$
$$\boldsymbol{\lambda} = (3, 2)\,. \tag{S14}$$

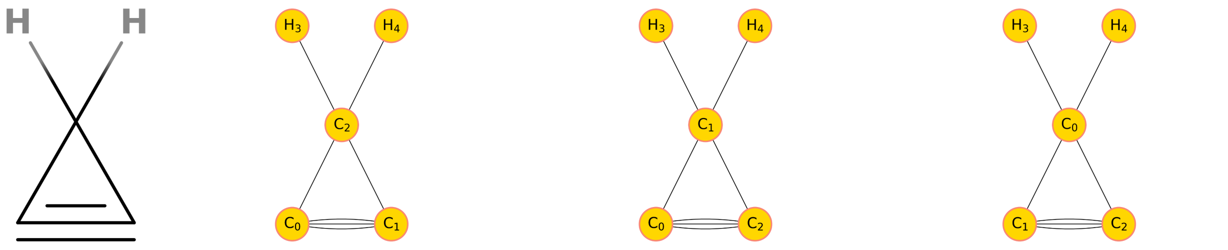

$$\mathbf{A}_1 = \begin{array}{c} \\ \end{array}\begin{pmatrix} 0 & 3 & 1 & 0 & 0 \\ 3 & 0 & 1 & 0 & 0 \\ 1 & 1 & 0 & 1 & 1 \\ 0 & 0 & 1 & 0 & 0 \\ 0 & 0 & 1 & 0 & 0 \end{pmatrix} \qquad \mathbf{A}_2 = \begin{pmatrix} 0 & 1 & 3 & 0 & 0 \\ 1 & 0 & 1 & 1 & 1 \\ 3 & 1 & 0 & 0 & 0 \\ 0 & 1 & 0 & 0 & 0 \\ 0 & 1 & 0 & 0 & 0 \end{pmatrix} \qquad \mathbf{A}_3 = \begin{pmatrix} 0 & 1 & 1 & 1 & 1 \\ 1 & 0 & 3 & 0 & 0 \\ 1 & 3 & 0 & 0 & 0 \\ 1 & 0 & 0 & 0 & 0 \\ 1 & 0 & 0 & 0 & 0 \end{pmatrix}$$

(Rows and columns of each matrix are labeled C0, C1, C2, H3, H4.)

Figure S2: Illustration of molecular graph isomorphism. Left: A constitutional isomer of C_3H_2. Right: The three possible isomorphic molecular graphs for the molecule on the left, including the corresponding adjacency matrices. The permutations to show isomorphism between the matrix pairs are $\boldsymbol{A}_2 = \boldsymbol{A}_1(1,2)$, $\boldsymbol{A}_3 = \boldsymbol{A}_1(0,1)(1,2)$, and $\boldsymbol{A}_3 = \boldsymbol{A}_2(0,1)$. Using the lexicographical ordering, it can be seen that $\mathbf{A}_1 > \mathbf{A}_2 > \mathbf{A}_3$. Consequently, $\mathbf{A}_1$ is canonical, whereas $\mathbf{A}_2$ and $\mathbf{A}_3$ are not.

S1.3 Filling Algorithm to Enumerate Adjacency Matrices

Enumerating all the unique constitutional isomers of a given molecular formula amounts to finding all the canonical adjacency matrices associated with this formula. An outline of the orderly enumeration scheme proposed by Grund [1] is provided in Algorithm 1.

In order to define the two functions `FindMaxEntry` and `DecreasePossible` used in Algorithm 1, three matrices are introduced. The matrix $\mathbf{M}$ is defined as [1]

$$\mathbf{M} := (M_{i,j})_{0 \leq i,j \leq N-1}\,, \qquad M_{i,j} = \begin{cases} \min(d_i, d_j) & d_i \neq d_j \\ d_i - 1 & d_i = d_j, i \neq j \\ 0 & i = j \end{cases}. \tag{S15}$$

The entry $M_{i,j}$ corresponds to the maximum value that $A_{i,j}$ can have, such that the valences of the atoms a_i and a_j are not exceeded, and at least one of the two atoms can be connected to one or more other atoms. Note that diatomic molecules represent an exception, and are handled explicitly in *enu* (with $M_{0,1} = d_0 = d_1$).

In *enu*, a slightly modified version of this matrix is used. For a given atom vector, the *degree of unsaturation* can be calculated as [6]

$$d_{\text{unsat}} = 1 + \frac{1}{2}\left(\sum_{k=0}^{K} \lambda_k(\delta_k - 2)\right), \tag{S16}$$

Algorithm 1: Filling Algorithm

```
// The two functions IncreaseIndex and DecreaseIndex can be found in Algorithm 2;
// FindMaxEntry and DecreasePossible are defined in Algorithm 3 and Algorithm 4

// start orderly enumeration with a ForwardStep at the first element of the matrix
ForwardStep (0,0)

Function ForwardStep(i,j):
    IncreaseIndex (i,j)
    if FindMaxEntry (i,j,x) then
        set A_{i,j} to x
        if i == N − 2 then
            // last index of the matrix is reached ⇒ potential adjacency matrix is complete
            Print(current matrix)
            // continue search for next potential adjacency matrix by back stepping
            BackwardStep (i,j)
        else
            // continue filling the matrix
            ForwardStep (i,j)
    else
        // no viable entry was found at the current matrix position
        BackwardStep (i,j)

Function BackwardStep(i,j):
    if j == 1 then
        // algorithm has terminated
        return
    else
        DecreaseIndex (i,j)
        if DecreasePossible (i,j) then
            // decrease current matrix entry by 1 and continue with a ForwardStep
            set A_{i,j} to A_{i,j} − 1
            ForwardStep(i,j)
        else
            // continue backstepping until an entry is found that can be decreased
            BackwardStep(i,j)
```

where δ_k is the valence of atom type α_k. It also holds that [6]

$$d_{\text{unsat}} = n_{\text{db}} + 2 \cdot n_{\text{tb}} + n_{\text{ring}}\,, \tag{S17}$$

where n_{db} is the number of double bonds, n_{tb} the number of triple bonds, and n_{ring} the number of rings in the molecule. Consequently, the maximum possible bond degree in a molecule is equal to $d_{\max} = 1 + d_{\text{unsat}}$. For this reason, an additonal restriction on $\mathbf{M}$ can be introduced as

$$M_{i,j} = \begin{cases} \min(d_i, d_j, d_{\max}) & d_i \neq d_j \\ \min(d_i - 1, d_{\max}) & d_i = d_j, i \neq j \\ 0 & i = j \end{cases}. \tag{S18}$$

This restriction is useful for molecules with low degrees of unsaturation; considering atoms with valences ≤ 4, it results in no gain as soon as $d_{\max} \geq 3$.

Algorithm 2: Methods to increase and decrease indices

```
Function IncreaseIndex(i,j):
    if j == (N − 1) then
        i ++
        j = i +1
    else
        j ++

Function DecreaseIndex(i,j):
    if j == i +1 then
        i −−
        j = N − 1
    else
        j −−
```

The two upper triangular matrices **L** and **C** are defined as [1]

$$L := (L_{i,j})_{0\leq i<j\leq N-1}\,, \qquad L_{i,j} := \min\left(d_i, \sum_{s=j+1}^{N-1} M_{i,s}\right) \tag{S19}$$

$$C := (C_{i,j})_{0\leq i<j\leq N-1}\,, \qquad C_{i,j} := \min\left(d_j, \sum_{s=i+1}^{N-1} M_{s,j}\right) \tag{S20}$$

An entry $L_{i,j}$ corresponds to the maximum possible *row capacity* after position (i, j), *i.e.*, the maximum number of potential bonds atom a_i can form with the atoms $a_{j+1}, a_{j+2}, \ldots, a_{N-1}$. Analogously, an entry $C_{i,j}$ corresponds to the maximum possible *column capacity* after position (i, j).

Additionally, the values $\hat{L}_{i,j}$ and $\hat{C}_{i,j}$ are defined as [1]

$$\hat{L}_{i,j} := d_i - \sum_{s=0}^{j-1} A_{i,s} \tag{S21}$$

$$\hat{C}_{i,j} := d_j - \sum_{s=0}^{i-1} A_{s,j}\,. \tag{S22}$$

$\hat{L}_{i,j}$ corresponds to the number of bonds that still have to be formed by atom a_i (including and after position (i, j)) such that it is fully connected, and $\hat{C}_{i,j}$ corresponds to the number of bonds that still have to be formed by atom a_j (including and after position (i, j)).

Using these definitions, one may now determine what constitutes a viable matrix element at a position (i, j). Four conditions have to be met by a potential matrix element x at (i, j) in the forward step [1]

$$x \geq 0 \tag{S23}$$

$$x \leq \min\{\hat{L}_{i,j}, \hat{C}_{i,j}, M_{i,j}\} \tag{S24}$$

$$x \geq \hat{L}_{i,j} - L_{i,j} \tag{S25}$$

$$x \geq \hat{C}_{i,j} - C_{i,j}\,. \tag{S26}$$

The two last condition ensure that x is sufficiently large for the atoms a_i and a_j to be both saturated in their respective valences once the corresponding row/column is filled. For example, for $i = N - 2$ and

$j = N-1$, if $a_i = a_{N-2}$ (*i.e.*, the second to last atom in the atom vector) is a carbon atom with valence four that is already singly-bonded to one of the atoms a_0 to a_{j-1}, then $\hat{L}_{i,j} = 3$ (*i.e.* a_i still needs to form three bonds to be saturated in its valence). $L_{i,j} = 0$ (*i.e.*, the row capacity after $(i,j) = (N-2, N-1)$ is zero, since the row is complete). Then we have $\hat{L}_{i,j} - L_{i,j} = 3 - 0 = 3$, *i.e.* in order to saturate a_i in its valence, there would need to be a bond of at least degree three between atoms a_i and a_j. If the last atom in the atom vector, $a_j = a_{N-1}$, is *e.g.* a chlorine atom with valence one, this is not possible due to the restriction $M_{N-1,N-2} = 1$.

Analogously, during the backward step, an entry may be decreased by one if

$$A_{i,j} - 1 \geq 0 \tag{S27}$$

$$(A_{i,j} - 1) \geq \hat{L}_{i,j} - L_{i,j} \tag{S28}$$

$$(A_{i,j} - 1) \geq \hat{C}_{i,j} - C_{i,j}\,. \tag{S29}$$

Note that $A_{i,j} - 1$ is the new potential matrix element at position (i,j) and that Eqs. S25 and S26 are identical to Eqs. S28 and S29, respectively, when setting x to $A_{i,j} - 1$. This ensures that the current row/column can still be saturated after the backward step.

Given the above restrictions, the routines `findMaxEntry` and `decreasePossible` used by the forward and backward steps, respectively, are outlined in Algorithm 3 and Algorithm 4.

Algorithm 3: findMaxEntry

```
Function FindMaxEntry(i,j,x):
    x = min(L̂_{i,j}, Ĉ_{i,j}, M_{i,j})
    if x ≥ max(0, L̂_{i,j} − L_{i,j}, Ĉ_{i,j} − C_{i,j}) then
        return true
    else
        return false
```

Algorithm 4: decreasePossible

```
Function DecreasePossible(i,j):
    x = A_{i,j} − 1
    if x >= 0 and L̂_{i,j} − x <= L_{i,j} and Ĉ_{i,j} − x <= C_{i,j} then
        return true
    else
        return false
```

S1.4 Connectivity Test

Algorithm 5 shows the connectivity test employed in the isomer enumerator.

Its application can be illustrated with a simple example for the disconnected molecular graphs depicted in Figure S3 The connectivity test proceeds as follows

- initialize: `visited = [1,0,0,0,0,0,0,0]`, `stack={0}`
- select last vertex in `stack`: `0`
  - add unvisited neighbors of `0` to `stack` and set corresponding entry in `visited` to `1`

Algorithm 5: Connectivity Test

```
create a last-in-first-out stack that contains the first vertex
create a boolean vector visited, where each entry corresponds to a vertex
set the first entry of visited to true, all others to false
while stack not empty do
    select top vertex in stack
    for each neighbour of current vertex do
        if neighbour has not yet been visited then
            set visited at position of neighbour to true
            add neighbour to the stack
for each vertex do
    if vertex has not been visited then
        return false
return true
```

  - unvisited neighbors are `1` and `3`
  - `stack={1,3}`, `visited=[1,1,0,1,0,0,0,0]`
- select last vertex in `stack`: `3`
  - add unvisited neighbors of `3` to `stack` and set corresponding entry in `visited` to `1`
  - `3` does not have any unvisited neighbors
  - `stack = {1}`, `visited=[1,1,0,1,0,0,0,0]`
- select last vertex in `stack`: `1`
  - add unvisited neighbors of `1` to `stack` and set corresponding entry in `visited` to `1`
  - `1` does not have any unvisited neighbors
  - `stack = {}`, `visited=[1,1,0,1,0,0,0,0]`
- stack is empty and some entries in `visited` are still `0` $\Rightarrow$ the graph is not connected

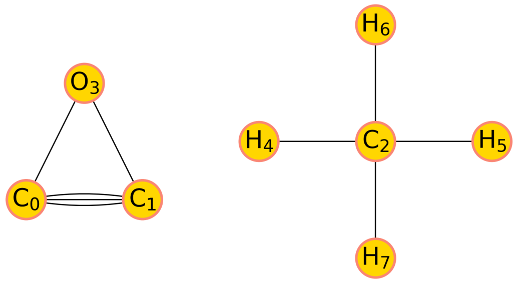

$$\mathbf{A} = \begin{pmatrix} 0 & 3 & 0 & 1 & 0 & 0 & 0 & 0 \\ 3 & 0 & 0 & 1 & 0 & 0 & 0 & 0 \\ 0 & 0 & 0 & 0 & 1 & 1 & 1 & 1 \\ 1 & 1 & 0 & 0 & 0 & 0 & 0 & 0 \\ 0 & 0 & 1 & 0 & 0 & 0 & 0 & 0 \\ 0 & 0 & 1 & 0 & 0 & 0 & 0 & 0 \\ 0 & 0 & 1 & 0 & 0 & 0 & 0 & 0 \\ 0 & 0 & 1 & 0 & 0 & 0 & 0 & 0 \end{pmatrix}$$

Figure S3: Example of a disconnected molecular graph for $\boldsymbol{\alpha} = (\mathrm{C}, \mathrm{O}, \mathrm{H})$, $\boldsymbol{\delta} = (4, 2, 1)$, and $\boldsymbol{\lambda} = (3, 1, 4)$ with the corresponding adjacency matrix $\boldsymbol{A}$.

S1.5 Canonicity Test

S1.5.1 Permutation Trees

To test a matrix for canonicity, the permutations $\pi \in S_{\boldsymbol{\lambda}}$ need to be available. These permutations can be systematically represented using a *permutation tree* [1, 7]. The permutation tree for a given molecular formula with N atoms is a tree with height N and $\lambda_0! \cdot \lambda_1! \cdot \ldots \cdot \lambda_K!$ leaves. The nodes of the tree are transpositions (swaps) of index pairs (i, j) $(0 \leq i \leq j \leq N-1)$. Recalling that the permutations are sorted, an index can only be swapped with a higher index in the same partition (see Equation S4). Thus, for a given index $i \in p_k$, the possible swaps are

$$(i, i), (i, i+1), (i, i+2), \ldots, (i, i+\lambda_k - 1) . \tag{S30}$$

These tuples with the first element set to i are the children of the nodes with depth i in the permutation tree. Consequently, the subtree of a node (i, j) is identical to the subtree of a node (i, j'). For example, the nodes with depth 1 (*i.e.* the children of the root) are all of the form $(0, j)$, and the nodes with depth N (*i.e.* the leaves of the tree) are all of the form $(N-1, N-1)$. Figure S4 provides an example of such a permutation tree.

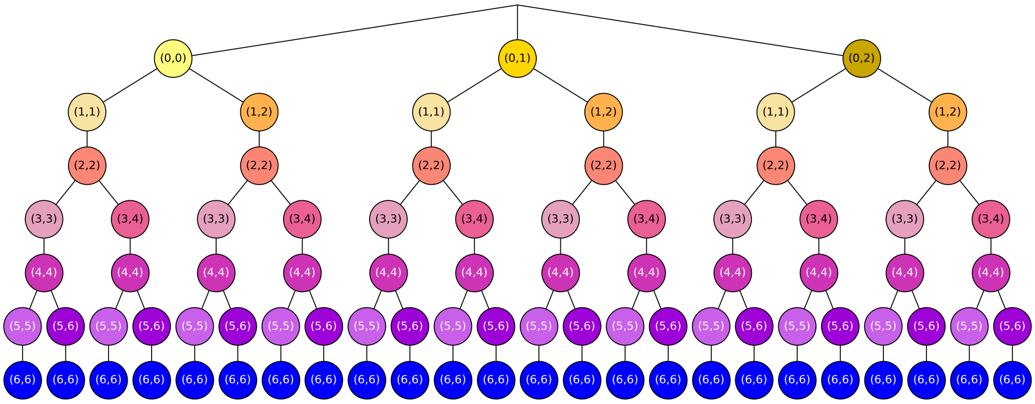

Figure S4: Example of a permutation tree. This figure shows the permutation tree for a molecular graph with $\boldsymbol{\lambda} = (3, 2, 2)$. Nodes that contain the same transposition are displayed in the same color.

A path from the root of the tree to a leaf defines a unique permutation $\pi \in S_{\boldsymbol{\lambda}}$ by combining the encountered nodes into a chain of index transpositions that are applied successively. The set of all possible paths from the root to the leaves spans the entire set $S_{\boldsymbol{\lambda}}$. Consequently, a depth-first traversal of the tree is a systematic way of listing all possible permutations in $S_{\boldsymbol{\lambda}}$.

S1.5.2 Representation Systems

The permutation trees introduced above permit to visualize the group $S_{\boldsymbol{\lambda}}$. However, even for a small $S_{\boldsymbol{\lambda}}$ such a tree would quickly become too large to store explicitly. A more convenient method for storing permutation trees relies on the use of a *representation system*. For a molecular graph with N atoms, such a system consists of N ordered sets, where the ith set contains the allowed permutations of atom a_i.

For example, the representation system of the permutation tree shown in Figure S4 reads:

(0,0), (0,1), (0,2)
(1,1), (1,2)
(2,2)
(3,3), (3,4)
(4,4)
(5,5), (5,6)
(6,6)

The algorithm used in *enu* to achieve a depth-first traversal of the permutation tree given its representation system is shown in Algorithm 6. The vector `current_permutation` is used to to indicate the current permutation in the traversal. This vector of integers has the same size N as the representation system. For example, for the above representation system, the vector `current_permutation = (2,0,0,0,0,1,0)` corresponds to the permutation $(0,2)(1,1)(2,2)(3,3)(4,4)(5,6)(6,6)$. The next permutation found by Algorithm 6 will be $(0,2)(1,1)(2,2)(3,4)(4,4)(5,5)(6,6)$, represented by `current_permutation = (2,0,0,1,0,0,0)`. A key advantage of this implementation is that it is very easy to skip subtrees of the permutation tree by changing the value of `cur_index` before the while-loop as desired (instead of setting it to $N-1$). This becomes important when pruning permutation trees (see Sec. S1.5.5).

Algorithm 6: Traversal of Representation System

```
// current_permutation represents the current permutation.
// cur_index stores the index of the last element that was changed in current_permutation
// N is the size of the representation system

// The function returns true if a next permutation was found (i.e.  we are not yet at
// the last permutation of the traversal) and stores the next permutation
// using current_permutation.
// The function returns false if there are no more permutations.
Function GetNextPermutation(const representation_system, current_permutation, N):
    cur_index = N −1
    while cur_index ≥ 0 do
        if current_permutation [cur_index ] + 1 < size (representation_system [cur_index ]) then
            current_permutation [cur_index ]++
            return true
        else
            current_permutation [cur_index ] = 0
            cur_index −−
    return false
```

S1.5.3 Naïve Canonicity Test

Given the representation system for the permutation tree of a molecular graph, the canonicity test for a given adjacency matrix **A** could be carried out in a brute-force fashion by performing a depth-first traversal of the tree and applying every possible permutation $\pi \in S_{\lambda}$ to **A**. An adjacency matrix **A** is rejected if at some point, a permutation $\pi \in S_{\lambda}$ is found for which $\mathbf{A}\pi > \mathbf{A}$. However, even for molecular formulas with only a small number of atoms and few isomers, the filling algorithm generates many potential adjacency matrices that have to be tested for canonicity before they are rejected. Additionally, the number of permutations in S_{λ} can become huge even for small molecules. Taking C_3H_8 as an example, the filling algorithm creates 80 potential adjacency matrices. Among these, 40 represent connected molecular graphs

and have to be tested for canonicity. There are $|S_{\{3,8\}}| = 3! \cdot 8! = 241\,920$ possible permutations that have to be applied to each of these matrices. At the end, only one of these adjacency matrices is canonical, the one corresponding to the canonical representation of propane. In the case of C_4H_{10}, there are 317 potential adjacency matrices, 81 of which are connected, and $|S_{\{4,10\}}| = 4! \cdot 10! = 87\,091\,200$ possible permutations. Here, two matrices are canonical, the ones corresponding to the canonical representations of *n*-butane and of isobutane. Both the number of adjacency matrices and the number of possible permutations increase exponentially with the number of atoms, making such a brute force canonicity test unusable [1].

S1.5.4 Blockwise Canonicity Test

Grund proposed a more efficient canonicity test [1]. For a given label vector $\boldsymbol{\alpha}$ and a corresponding partition vector $\boldsymbol{\lambda}$ of size $K+1$, the potential adjacency matrices can be subdivided into $K+1$ blocks of sizes $\lambda_0, \dots, \lambda_K$, respectively. The different blocks of an adjacency matrix $\mathbf{A}$ are denoted by $\mathbf{A}^{(0)}, \mathbf{A}^{(1)}, \dots, \mathbf{A}^{(K)}$, where the *k-th* block is defined as [1],

$$\mathbf{A}^{(k)} := (A_{i,j}^{(k)}) \qquad i,j \in p_k, p_{k+1}, \dots, p_K \ \wedge \ (i \in p_k \ \vee \ j \in p_k), \ 0 \le k \le K \tag{S31}$$

In plain words, the *k-th* block describes the bonds of the atoms of the same type α_k with each other, as well as with the atoms of types α_l with $l > k$, *i.e.*, that come after α_k in the label vector. As an example, the three blocks of the adjacency matrix in Main Text Figure 1 for $C_3O_2H_4$ can be illustrated as

$$\mathbf{A} = \begin{pmatrix} 0 & 1 & 2 & 0 & 1 & 0 & 0 & 0 & 0 \\ 1 & 0 & 0 & 0 & 0 & 1 & 1 & 1 & 0 \\ 2 & 0 & 0 & 2 & 0 & 0 & 0 & 0 & 0 \\ 0 & 0 & 2 & 0 & 0 & 0 & 0 & 0 & 0 \\ 1 & 0 & 0 & 0 & 0 & 0 & 0 & 0 & 1 \\ 0 & 1 & 0 & 0 & 0 & 0 & 0 & 0 & 0 \\ 0 & 1 & 0 & 0 & 0 & 0 & 0 & 0 & 0 \\ 0 & 1 & 0 & 0 & 0 & 0 & 0 & 0 & 0 \\ 0 & 0 & 0 & 0 & 1 & 0 & 0 & 0 & 0 \end{pmatrix}. \tag{S32}$$

Since the filling algorithm constructs the matrices row by row, and the symmetric entries are set simultaneously, it also fills them blockwise [1]. This means that a partially filled adjacency matrix can already be tested for canonicity within the filled blocks. Since the definition of the lexicographical order of two matrices depends on the first unequal element, if the already filled block can be permuted to a lexicographically larger form, the current filling of the blocks can be immediately stopped as it will never lead to a canonical matrix.

This approach is included in the filling algorithm by adjusting the `ForwardStep` function. Whenever a viable matrix entry x is found in the forward step, and the current index (i, j) is the final index of a block, the matrix can already be tested for canonicity. If the matrix is canonical at that point, the next forward step is called. Otherwise, the algorithm continues with a backward step.

In addition to detecting non-canonical matrices early, a canonicity test on a partially filled matrix is less time consuming, since the lexicographical ordering only has to be checked for the current block (since the previous blocks have already been tested). Nevertheless, it still involves processing all permutations of $S_{\boldsymbol{\lambda}}$ (at least until a lexicographically larger adjacency matrix is found).

S1.5.5 Pruning the Permutation Tree: Stabilizers

To further improve the performance of the canonicity test, the concept of *stabilizers* is introduced. The stabilizer of the blocks $0,\dots,k$ is defined as the set [1].

$$Aut^{(k)}(\mathbf{A}) := \left\{ \pi \in S_{\boldsymbol{\lambda}} : \mathbf{A}^{(0)} = \mathbf{A}^{(0)}\pi, \dots, \mathbf{A}^{(k)} = \mathbf{A}^{(k)}\pi \right\} \subseteq S_{\boldsymbol{\lambda}}\,. \tag{S33}$$

In plain words, the representation system of the stabilizer set $Aut^{(k)}(\mathbf{A})$ consists of all permutations in $S_{\boldsymbol{\lambda}}$ which leave the blocks $0,\dots,k$ of $\mathbf{A}$ identical upon application. The set $Aut(\mathbf{A}) := Aut^{(K)}(\mathbf{A})$ is the so-called *automorphism group* of the entire matrix.

Clearly, it must hold that, for a given matrix $\mathbf{A}$ [1]

$$Aut \equiv Aut^{(K)} \subseteq Aut^{(K-1)} \subseteq \cdots \subseteq Aut^{(k)} \subseteq \cdots \subseteq Aut^{(0)} \subseteq S_{\boldsymbol{\lambda}} \tag{S34}$$

As previously discussed, whenever a new block $k+1$ is filled, the previous blocks up to k were already checked to be canonical. Thus, it holds that for $0 \leq l \leq k$,

$$\mathbf{A}^{(l)} \geq \mathbf{A}^{(l)}\pi \quad \forall \pi \in S_{\boldsymbol{\lambda}}\,. \tag{S35}$$

Due to the restrictions on the permutations in $S_{\boldsymbol{\lambda}}$, this must remain true after block $k+1$ is filled. Obviously, the only permutations that still have the potential to produce a lexicographically larger adjacency matrix in the blocks $0,\dots,k+1$ are the ones that leave the blocks up to k identical, as all the other permutations lead to an adjacency matrix which is smaller within the blocks $0,\dots,k$. These permutations correspond to the ones contained in the stabilizer $Aut^{(k)}$ [1].

Taking this observation into account, whenever a new block $k+1$ is filled and the matrix is to be tested for canonicity, it is sufficient to test the new block $k+1$, and the canonicity test only needs to consider the permutations in $\pi \in Aut^{(k)}$. A block $k+1$ is thus canonical if [1]

$$\mathbf{A}^{(k+1)} \geq \mathbf{A}^{(k+1)}\pi \quad \forall \pi \in Aut^{(k)}(\mathbf{A})\,. \tag{S36}$$

In order to perform a canonicity test of block $(k+1)$, $Aut^{(k)}$ is required. The straightforward approach for obtaining this stabilizer is to produce it during the canonicity test of block k. Since the permutations $\pi \in Aut^{(k-1)}$ are used for the canonicity test of this block, and a lexicographical comparison between the matrices $\mathbf{A}$ and $\mathbf{A}\pi$ has to be performed, one simply has to find all the permutations $\pi \in Aut^{(k-1)}$ for which

$$\mathbf{A}^{(k)} = \mathbf{A}^{(k)}\pi\,. \tag{S37}$$

Once a stabilizing permutation π is found at a node (i,j) during the depth-first traversal of the permutation tree, it holds that [1]

$$\mathbf{A}^{(k)} \geq \mathbf{A}^{(k)}\pi' \tag{S38}$$

for all permutations π' found by traversing the subtree of node (i,j). Due to the depth-first traversal of the permutation tree, and since the current permutation is a stabilizing one, the permutations found by traversing this subtree were all already tested during the current canonicity check. Since the canonicity test is still ongoing, the current matrix was not (yet) found to not be canonical. Thus, any permutation that is encountered in the subtree of the current stabilizing permutation cannot lead to a lexicographically larger adjacency matrix. Finding the first stabilizing permutation during every part of the depth-first

traversal is thus sufficient to test the canonicity of a matrix block. These first encountered stabilizing permutations form the representation system of the stabilizer $Aut^{(k)}$. Once such a permutation is found, it is added to the representation system and further traversal of the current subtree can be stopped. When block $k+1$ is checked for canonicity, only the permutations formed by the representation system of $Aut^{(k)}$ have to be considered.

Consider the permutation tree in Figure S4. Let us assume we find that the permutation $(0,1)$ is a stabilizer of block zero. When we encounter this permutation, we already checked the permutations $(5,6)$, $(3,4)$, $(3,4)(5,6)$, $(1,2)$, $(1,2)(5,6)$, $(1,2)(3,4)$, and $(1,2)(3,4)(5,6)$. All of these permutations must have lead to an adjacency matrix that is either smaller or equal to the current one (otherwise the current matrix would already have been rejected). Since the permutation $(0,1)$ is a stabilizer and thus leaves the adjacency matrix unchanged, checking the subtree of permutation $(0,1)$, i.e. the permutations $(0,1)(5,6)$, $(0,1)(3,4)$, $(0,1)(3,4)(5,6)$, $(0,1)(1,2)$, $(0,1)(1,2)(5,6)$, $(0,1)(1,2)(3,4)$, and $(0,1)(1,2)(3,4)(5,6)$ is guaranteed to also produce only adjacency matrices that are either smaller or equal to the current one. Finally, if the encountered stabilizers are, for example, $(3,4)(5,6)$ and $(0,1)$, the representation system for the stabilizers of block zero will be

$$
\begin{aligned}
&(0,0),\ (0,1)\\
&(1,1)\\
&(2,2)\\
&(3,3),\ (3,4)(5,6)\\
&(4,4)\\
&(5,5)\\
&(6,6)\quad .
\end{aligned}
$$

Therefore, the only permutations that need to be considered for the canonicity test of block one will be $(3,4)(5,6)$, $(0,1)$, and $(0,1)(3,4)(5,6)$.

Another helpful observation is that a stabilizer of a previous block is guaranteed to be a stabilizer of the current block, provided that it only affects rows/columns that are part of the previous block. For example, if $(0,1)$ is a stabilizer of block zero, this permutation does not actually have to be checked when we test block one for canonicity. It will swap rows zero and one as well as columns zero and one, which only has an effect on block zero. Since the permutation is a stabilizer of block zero, this leaves the matrix unchanged. The permutation $(0,1)$ can thus directly be added to the representation system of the stabilizers of block one and its subtree can be skipped.

S1.5.6 Semi-Canonicity

In addition to the central concept of canonicity, Grund uses the idea of *semi-canonicity* [1] as a weak canonicity criterion. If an adjacency matrix is not semi-canonical, it cannot be canonical, but if it is semi-canonical it is not necessarily canonical. It is based on determining a refined partitioning of the atom vector according to the bonds they form in $\mathbf{A}$. A row of the matrix is semi-canonical if the matrix elements within each of these refined partitions are decreasing. The major advantage of the semi-canonicity test is its relatively low computational cost compared to the full blockwise canonicity test described above. The semi-canonicity criterion can be used directly at every iteration of the forward step, instead of just blockwise. It introduces an additional restriction on the current matrix element (*i.e.*, it cannot be larger than the previous element if that element is in the same refined partition), potentially skipping many partially filled matrices that would otherwise be discarded at a later stage.

This provides an additional constraint on the choice of a new matrix entry in the forward step. The value set at a new entry cannot be larger than the previous value if they are in the same refined partition. This restriction is added to the function `findMatrixEntry` in the `ForwardStep`.

S1.5.7 Index Jumps

A fast canonicity test represents one opportunity to make the enumeration algorithm more efficient. Another useful trick is to skip as many non-canonical matrices as possible. Grund introduced a *canonical learning criterion*, which allows to skip the orderly generation of other non-canonical adjacency matrices when a non-canonical matrix is generated [1]. It is based on identifying the index of the first different element in the current non-canonical matrix and the lexicographically larger matrix that was found during the canonicity test.

S1.6 Special Treatment of Hydrogen Atoms

For a given label vector $\boldsymbol{\alpha} = (\alpha_0, \ldots, \alpha_{K-1}, \mathrm{H})$ and partition vector $\boldsymbol{\lambda} = (\lambda_0, \ldots, \lambda_{K-1}, \lambda_K)$, λ_K describes the number of hydrogen atoms in the molecular formula. The hydrogen vector can be defined as [1]

$$\mathbf{h} = (h_0, ..., h_{\hat{N}-1}), \tag{S39}$$

where

$$\hat{N} := \sum_{k=0}^{K-1} \lambda_k \tag{S40}$$

is the number of non-hydrogen atoms and

$$\sum_{i=0}^{\hat{N}-1} h_i = \lambda_K. \tag{S41}$$

In plain words, the length of the hydrogen vector is equal to the number of non-hydrogen atoms in the molecule, and its entries sum up to the number λ_K of hydrogen atoms in the molecule. This hydrogen vector will be used to describe a possible distribution of the hydrogen atoms in terms of their binding to the non-hydrogen atoms of the molecule.

Given the partition vector

$$\bar{\boldsymbol{\lambda}} = (\lambda_0, \lambda_1, \ldots, \lambda_{K-1}) \tag{S42}$$

of the non-hydrogen atoms, two hydrogen vectors $\mathbf{h}$ and $\mathbf{h}'$ are equivalent if there exists a $\pi \in S_{\bar{\boldsymbol{\lambda}}}$ such that [1]

$$\mathbf{h}' = \mathbf{h}\pi. \tag{S43}$$

Analogous to the definition for adjacency matrices, a hydrogen vector $\mathbf{h}$ is said to be *canonical* if

$$\mathbf{h} \geq \mathbf{h}\pi \quad \forall \pi \in S_{\bar{\boldsymbol{\lambda}}}, \tag{S44}$$

which is equivalent to the condition that the entries of $\mathbf{h}$ are decreasing within the partitions described by $\bar{\boldsymbol{\lambda}}$. [1] For a given canonical hydrogen vector, an adapted atom vector

$$\hat{\boldsymbol{a}} = (a_0\mathrm{H}_{\mathrm{h}_0}, a_1\mathrm{H}_{\mathrm{h}_1}, ..., a_{\hat{N}-1}\mathrm{H}_{\mathrm{h}_{\hat{N}-1}}) \tag{S45}$$

can be defined, with a corresponding adapted degree vector

$$\hat{\mathbf{d}} = (d_0 - h_0, d_1 - h_1, ..., d_{\hat{N}-1} - h_{\hat{N}-1}) \,. \tag{S46}$$

Due to the definition of canonicity for the hydrogen vector, atoms with the same label and the same number of implicit hydrogen atoms are always listed next to each other. A new partition vector

$$\hat{\boldsymbol{\lambda}} = (\hat{\lambda}_0, \hat{\lambda}_1, \ldots, \hat{\lambda}_{\hat{K}}) \tag{S47}$$

can be defined, which counts the number of occurrences of each group of atoms with the same label and the same number of implicit hydrogen atoms. For the newly introduced partition vectors it must hold that

$$\prod_{k=0}^{K} \lambda_k! \geq \prod_{i=0}^{\hat{K}} \hat{\lambda}_k! \tag{S48}$$

and thus the number of permutations in $S_{\hat{\boldsymbol{\lambda}}}$ is (often considerably) smaller than the number of permutations in $S_{\boldsymbol{\lambda}}$. Here, $\hat{K}$ is the number of different atom types in the atom vector when taking into account the distribution of the hydrogen atoms.

With this, the enumeration process can be made more efficient. Instead of using the filling algorithm directly, the algorithm performs an orderly enumeration of canonical hydrogen vectors. The filling algorithm is then used to create canonical adjacency matrices for each of these hydrogen vectors using the corresponding input vectors $\hat{\boldsymbol{a}}$ and $\hat{\boldsymbol{d}}$.

The algorithm that was used to produce canonical hydrogen vectors in this project is adapted from the orderly enumeration algorithm in Algorithm 1, and also consists of a forward step, a backward step and a canonicity test. Analogous to the matrix $\mathbf{M}$, defined in Equation S18, a vector $\mathbf{m}$ is defined as

$$\mathbf{m} = (m_0, m_1, \ldots, m_{\hat{N}-1}) \,, \qquad m_i = \min(d_i - 1, \lambda_K) \,, \tag{S49}$$

such that each entry corresponds to the maximum number of hydrogen atoms that can be connected to the corresponding atom in the atom vector. A non-hydrogen atom has to have at least one bond left to connect to another non-hydrogen atom, and it cannot be connected to more hydrogen atoms than the total number of hydrogen atoms λ_K in the molecular formula. The algorithm is shown in Algorithm 7. Note that the case that only one non-hydrogen atom is present (*e.g.* C_1H_4) is an exception that must be handled explicitly in the program.

The following example illustrates the dramatic impact of this approach on the computational cost even for small molecules. For the molecule $C_3O_2H_4$, the following canonical hydrogen vectors can be constructed

$$\begin{aligned}
\mathbf{h}_0 &= (3,1,0,0,0) \\
\mathbf{h}_1 &= (3,0,0,1,0) \\
\mathbf{h}_2 &= (2,2,0,0,0) \\
\mathbf{h}_3 &= (2,1,1,0,0) \\
\mathbf{h}_4 &= (2,1,0,1,0) \\
\mathbf{h}_5 &= (2,0,0,1,1) \\
\mathbf{h}_6 &= (1,1,1,1,0) \\
\mathbf{h}_7 &= (1,1,0,1,1)
\end{aligned} \tag{S50}$$

Algorithm 7: Filling Algorithm for the Hydrogen Vector

```
// start the algorithm by calling a HForwardStep at index -1
initialize empty hydrogen vector h
HForwardStep (-1)

Function HForwardStep(i):
    i ++
    if i == N̂ then
        if sum (h) == λ_K then
            // h is completely filled, and the sum over h is equal to the number
            // of hydrogen atoms in the given molecular formula
            HCanonicityTest(h)
        else
            // h is completely filled, but the sum over h is not equal to the number
            // of hydrogen atoms in the given molecular formula, and thus h is not
            // a viable hydrogen vector
            HBackwardStep(i)
    else
        // potential entry at i in h is the minimum of the maximum entry possible, given
        // by entry i of m, and the number of hydrogen atoms that are not yet distributed
        x = min(m_i, λ_K - sum (h))
        set h_i to x
        HForwardStep(i)


Function HBackwardStep(i,j):
    if i == 0 then
        // algorithm has terminated
        return
    i −−
    if h_i > 0 then
        // decrease current entry of h and test if the corresponding hydrogen vector h
        // is now canonical up to i, i.e.  if the entries are decreasing within their
        // partitions up to i
        set h_i to h_i-1
        if h is canonical up to index i then
            HForwardStep(i)
        else
            i ++
            HBackwardStep (i)
    else
        HBackwardStep(i)
```

with the corresponding atom vectors

$$
\begin{aligned}
\hat{\boldsymbol{a}}_0 &= (\mathrm{CH_3}, \mathrm{CH_1}, \mathrm{C}, \mathrm{O}, \mathrm{O}) \\
\hat{\boldsymbol{a}}_1 &= (\mathrm{CH_3}, \mathrm{C}, \mathrm{C}, \mathrm{OH_1}, \mathrm{O}) \\
\hat{\boldsymbol{a}}_2 &= (\mathrm{CH_2}, \mathrm{CH_2}, \mathrm{C}, \mathrm{O}, \mathrm{O}) \\
\hat{\boldsymbol{a}}_3 &= (\mathrm{CH_2}, \mathrm{CH_1}, \mathrm{CH_1}, \mathrm{O}, \mathrm{O}) \\
\hat{\boldsymbol{a}}_4 &= (\mathrm{CH_2}, \mathrm{CH_1}, \mathrm{C}, \mathrm{OH_1}, \mathrm{O}) \\
\hat{\boldsymbol{a}}_5 &= (\mathrm{CH_2}, \mathrm{C}, \mathrm{C}, \mathrm{OH_1}, \mathrm{OH_1}) \\
\hat{\boldsymbol{a}}_6 &= (\mathrm{CH_1}, \mathrm{CH_1}, \mathrm{CH_1}, \mathrm{OH_1}, \mathrm{O}) \\
\hat{\boldsymbol{a}}_7 &= (\mathrm{CH_1}, \mathrm{CH_1}, \mathrm{C}, \mathrm{OH_1}, \mathrm{OH_1})\,.
\end{aligned}
\tag{S51}
$$

and degree vectors

$$\begin{aligned}
\hat{\mathbf{d}}_0 &= (1,3,4,2,2) \\
\hat{\mathbf{d}}_1 &= (1,4,4,1,2) \\
\hat{\mathbf{d}}_2 &= (2,2,4,2,2) \\
\hat{\mathbf{d}}_3 &= (2,3,3,2,2) \\
\hat{\mathbf{d}}_4 &= (2,3,1,1,2) \\
\hat{\mathbf{d}}_5 &= (2,4,4,1,1) \\
\hat{\mathbf{d}}_6 &= (3,3,3,1,2) \\
\hat{\mathbf{d}}_0 &= (3,3,4,1,1)\,.
\end{aligned} \tag{S52}$$

The corresponding label vectors are

$$\begin{aligned}
\hat{\boldsymbol{\alpha}}_0 &= (\mathrm{CH_3},\mathrm{CH_1},\mathrm{C},\mathrm{O}) \\
\hat{\boldsymbol{\alpha}}_1 &= (\mathrm{CH_3},\mathrm{C},\mathrm{OH_1},\mathrm{O}) \\
\hat{\boldsymbol{\alpha}}_2 &= (\mathrm{CH_2},\mathrm{C},\mathrm{O}) \\
\hat{\boldsymbol{\alpha}}_3 &= (\mathrm{CH_2},\mathrm{CH_1},\mathrm{O}) \\
\hat{\boldsymbol{\alpha}}_4 &= (\mathrm{CH_2},\mathrm{CH_1},\mathrm{C},\mathrm{OH_1},\mathrm{O}) \\
\hat{\boldsymbol{\alpha}}_5 &= (\mathrm{CH_2},\mathrm{C},\mathrm{OH_1},\mathrm{OH_1}) \\
\hat{\boldsymbol{\alpha}}_6 &= (\mathrm{CH_1},\mathrm{OH_1},\mathrm{O}) \\
\hat{\boldsymbol{\alpha}}_7 &= (\mathrm{CH_1},\mathrm{C},\mathrm{OH_1})\,.
\end{aligned} \tag{S53}$$

with the corresponding partition vectors

$$\begin{aligned}
\hat{\boldsymbol{\lambda}}_0 &= (1,1,1,2) \\
\hat{\boldsymbol{\lambda}}_1 &= (1,2,1,1) \\
\hat{\boldsymbol{\lambda}}_2 &= (2,1,2) \\
\hat{\boldsymbol{\lambda}}_3 &= (1,2,2) \\
\hat{\boldsymbol{\lambda}}_4 &= (1,1,1,1,1) \\
\hat{\boldsymbol{\lambda}}_5 &= (1,2,2) \\
\hat{\boldsymbol{\lambda}}_6 &= (3,1,1) \\
\hat{\boldsymbol{\lambda}}_7 &= (2,1,2)\,.
\end{aligned} \tag{S54}$$

The number of elements in the symmetric group for the new partition vectors are

$$
\begin{aligned}
|S_{\hat{\boldsymbol{\lambda}}_0}| &= 1! \cdot 1! \cdot 1! \cdot 2! = 2 \\
|S_{\hat{\boldsymbol{\lambda}}_1}| &= 1! \cdot 2! \cdot 1! \cdot 1! = 2 \\
|S_{\hat{\boldsymbol{\lambda}}_2}| &= 2! \cdot 1! \cdot 2! = 4 \\
|S_{\hat{\boldsymbol{\lambda}}_3}| &= 1! \cdot 2! \cdot 2! = 4 \\
|S_{\hat{\boldsymbol{\lambda}}_4}| &= 1! \cdot 1! \cdot 1! \cdot 1! \cdot 1! = 1 \\
|S_{\hat{\boldsymbol{\lambda}}_5}| &= 1! \cdot 2! \cdot 2! = 4 \\
|S_{\hat{\boldsymbol{\lambda}}_6}| &= 3! \cdot 1! \cdot 1! = 6 \\
|S_{\hat{\boldsymbol{\lambda}}_7}| &= 2! \cdot 1! \cdot 2! = 4
\end{aligned}
\tag{S55}
$$

which are all *considerably* smaller than the number of elements in the original symmetric group

$$
|S_{\boldsymbol{\lambda}}| = 3! \cdot 2! \cdot 4! = 288\,. \tag{S56}
$$

Note that the canonical ordering of the atom vector (according to the valence, size of the partition, and atomic number) is generated for each distribution of the hydrogens prior to starting the enumeration algorithm. For example, an oxygen atom without implicit hydrogen and with a valence of two would come before a carbon atom with three implicit hydrogens and a valence of one.

The specification of implicit hydrogens from user input, as described in the Main Text Section on Implementation Details, is simply a restriction of the allowed distributions of the hydrogens. If an atom is already assigned a specific number of implicit hydrogen atoms, this number remains constant in the hydrogen vector.

S1.7 Comparison to Brute-Force Approach

To assess the influence of the optimizations described in the previous sections on the performance of *enu*, we compared the timings for the enumeration of constitutional straight-chain alkane isomers when using the optimized code to using a brute-force approach (Table S1, Figure S5). For the brute-force approach, (*i*) the canonicity test is only performed once a complete adjacency matrix is found by the orderly enumeration (instead of a blockwise canonicity test); (*ii*) the semi-canonicity criterion is not used; and (*iii*) the canonical learning criterion is not used. Further, a comparison is made between the brute-force approach with and without the special treatment of the hydrogen atoms. The modified source code for the brute-force approach is also provided in the CombiFF GitHub repository in the *brute-force* branch (`https://github.com/csms-ethz/CombiFF/commit/10dc5e35cc3ee0d29d153951d70760511cc82e14`).

molecule	n_{consti}	$t_{optimized}$ [s]	$t_{brute\text{-}force}$ [s]	$t_{brute\text{-}force,hyd}$ [s]
C_1H_4	1	<0.01	<0.01	<0.01
C_2H_6	1	<0.01	<0.01	<0.01
C_3H_8	1	<0.01	<0.01	0.02
C_4H_{10}	2	<0.01	<0.01	11.52
C_5H_{12}	3	<0.01	<0.01	17173.02
C_6H_{14}	5	<0.01	<0.01	–
C_7H_{16}	9	<0.01	0.05	–
C_8H_{18}	18	<0.01	0.00	–
C_9H_{20}	35	0.00	0.12	–
$C_{10}H_{22}$	75	0.00	0.29	–
$C_{11}H_{24}$	159	0.00	2.08	–
$C_{12}H_{26}$	355	0.01	18.96	–
$C_{13}H_{28}$	802	0.02	193.26	–
$C_{14}H_{30}$	1858	0.05	2447.17	–
$C_{15}H_{32}$	4347	0.13	35110.38	–
$C_{16}H_{34}$	10359	0.31	–	–
$C_{17}H_{36}$	24894	0.93	–	–
$C_{18}H_{38}$	60523	2.74	–	–
$C_{19}H_{40}$	148284	8.22	–	–
$C_{20}H_{42}$	366319	25.55	–	–
$C_{21}H_{44}$	910726	79.41	–	–
$C_{22}H_{46}$	2278658	253.57	–	–
$C_{23}H_{48}$	5731580	834.42	–	–
$C_{24}H_{50}$	14490245	2895.93	–	–
$C_{25}H_{52}$	36797588	10040.19	–	–

Table S1: Comparison of the optimized code with a brute-force approach. The table shows the number n_{consti} of constitutional isomers of the straight-chain alkanes from C_1H_4 to $C_{25}H_{52}$, as well as the wall-clock time spent on their enumeration for three versions of the code: (*i*) the optimized code ($t_{optimized}$); (*ii*) a brute-force approach where hydrogens are treated implicitly ($t_{brute\text{-}force}$); and (*iii*) a brute-force approach where hydrogen atoms are treated explicitly ($t_{brute\text{-}force,hyd}$). All calculations were performed on AMD EPYC 7763 CPUs of the ETH Zürich Euler cluster [8] with the isomer enumerator's *count_only* option and averaged over five runs. Note that the brute-force calculations were stopped after exceeding 10^4 s. The results are visualized in Figure S5.

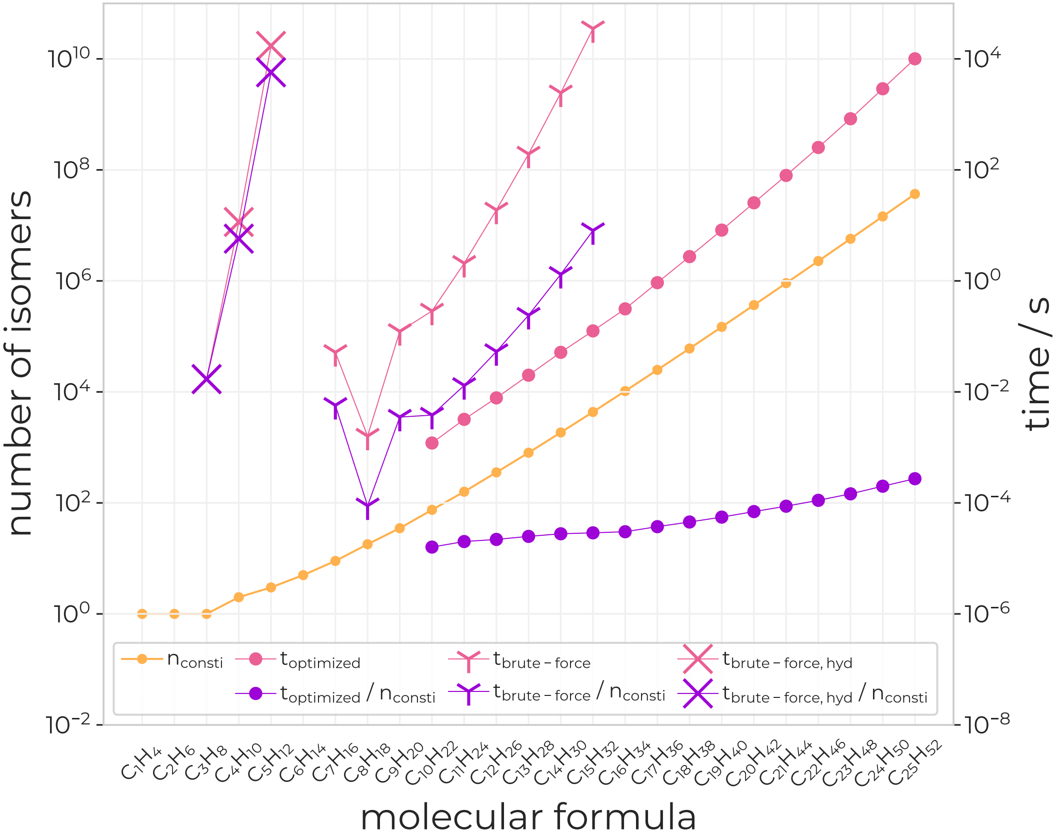

Figure S5: Comparison of the optimized code with a brute-force approach. The plot compares the performance of the isomer enumerator to a brute-force approach in the context of the straight-chain alkanes C_nH_{2n+2} from C_1H_4 to $C_{25}H_{52}$. The plot shows the number of constitutional alkane isomers depending on the number of carbon atoms. Further, it shows the wall-clock time to enumerate the isomers (pink) and the wall-clock time spent per isomer for this enumeration (purple) for three versions of the code: (*i*) the optimized code ($t_{\text{optimized}}$, dots); (*ii*) a brute-force approach where hydrogens are treated implicitly ($t_{\text{brute-force}}$, tri-downs); and (*iii*) a brute-force approach where hydrogen atoms are treated explicitly ($t_{\text{brute-force,hyd}}$, crosses). The left vertical axis shows the number of isomers and the right vertical axis shows the elapsed wall-clock time. All calculations were performed on AMD EPYC 7763 CPUs of the ETH Zürich Euler cluster [8] with the isomer enumerator's *count_only* option and averaged over five runs. Note that the brute-force calculations were stopped after exceeding 10^4 s and values with $t < 0.001$ s are not plotted. The numerical values are provided in Table S1.

S2 SMILES canonicalization

Both the algorithm proposed by Weininger [9] and the one proposed by Schneider [10] for canonicalization of SMILES strings start by assigning an initial atom ordering, considering atom *invariants*, which are based on the properties of the atoms in the molecule. The invariants used for the implementation of the canonicalization algorithm in *enu* are the ones proposed by Weininger et al. In order to create the atomic invariants, the following atomic properties are used: the number n_C of connections (*i.e.*, single or multiple bonds) to non-hydrogen atoms, the number n_B of bonds to non-hydrogen atoms, the atomic number Z, the sign of charge s (0 for zero or positive, 1 for negative), the net charge c (in units of e), and the number n_H of attached hydrogens [9]. Given these properties, the initial atom invariant is created by combining them into an eight digit number

$$10^7 \cdot n_C + 10^5 \cdot n_B + 10^3 \cdot Z + 10^2 \cdot s + 10 \cdot c + n_H \,. \tag{S57}$$

Considering the molecule shown in Main Text Figure 1 as an example, the initial atom invariants are shown in the second row of Table S2.

atoms	C_0	C_1	C_2	O_3	C_4	H_5	H_6	H_7	H_8
invariant	30406000	10106003	20406000	10208000	10108001	10101000	10101000	10101000	10101000
initial index (W)	5	1	4	3	2	0	0	0	0
initial index (S)	8	4	7	6	5	0	0	0	0

Table S2: initial invariants for the atoms of the molecule shown in Main Text Figure 1

Given these initial invariants, an initial ordering of the atoms is achieved by assigning indices in order of increasing value of the invariant. On one hand, Weininger et al. propose to directly assign consecutive indices. On the other hand, Schneider et al. propose a more elaborate and stable indexing that leaves sufficient space between the indices such that when an index is reassigned, no other index needs change. This is achieved by assigning the same index i_1 to all of the n_1 atoms with the same initial invariant, and then using the next higher index $i_2 = i_1 + n_1$ [10]. The two kinds of initial indices are shown in the last two rows of Table S2, respectively, and the one that is used in the *enu* program is the one developed by Schneider et al. The initial index assignment divides the atoms into different partitions, or *equivalence classes*, with all atoms that have the same index belonging to the same equivalence class [10]. Once the initial equivalence classes are created, they are continuously refined until each equivalence class consists of just one atom.

For two atoms a and a' possessing the same number of first neighbors with corresponding indices $i_0 > \cdots > i_{n_C-1}$ and $i'_0 > \cdots > i'_{n_C-1}$, a lexicographical ordering can be defined as

$$a > a' \Leftrightarrow \exists j : i_j > i'_j \wedge i_k = i'_k \quad \forall k < j \tag{S58}$$

and

$$a = a' \Leftrightarrow i_k = i'_k \quad \forall 0 \leq k \leq n_C - 1 \,. \tag{S59}$$

Due to the definition of the initial invariants, only atoms that have the same number n_C of neighbors can be in the same equivalence class. Thus, when refining an equivalence class, all the atoms in the corresponding partition are compared in a pairwise fashion and the index of the lexicographically larger atom is increased by one. If the two atoms are lexicographically equal, their respective indices are left

unchanged. Thus, the partitions that are newly created due to the index reassignments can still contain more than one atom.

The algorithm proposed by Schneider et al. keeps a list of the partitions that need to be refined, initially containing all equivalence classes that consist of more than one atom. It proceeds by always refining the equivalence class in the list that corresponds to the highest atom index. Once the refinement is finished, the corresponding partition is erased from the list. At the same time, all the equivalence classes that containing atoms whose neighbors are affected by the index reassignment of the last refinement step are added back to the list for reevaluation if they still contain more than one atom. The algorithm then continues with the refinement of the equivalence class in the list that corresponds to the currently highest atom index.

At some point, the list of partitions that need to be refined may become empty, although there are still equivalence classes containing more than one element. For this scenario, Schneider et al. propose a tie-breaking step. The tiebreaking is performed in the equivalence class that corresponds to the highest atom index that still consists of more than one atom. The atom with the largest original index within that equivalence class is assigned the highest possible index within this class. After this tie-breaking step, the refinement process is started once more. The refinement process and the tie-breaking step are used in alternance, until all equivalence classes consist of just one atom.

Note that while this algorithm produces a canonical atomic ordering in almost all cases, if the molecule is highly symmetrical, it is still possible that the final ordering is not unique but depends on the initial atom ordering. For this, Schneider et al. introduce two new invariants. In the *enu* program, these two additional invariants are not used since the canonical adjacency matrix provides an canonical initial atom ordering. Thus, the SMILES string is guaranteed to be canonical.

References

[1] Grund, R.: Konstruktion molekularer Graphen mit gegebenen Hybridisierungen und überlappungsfreien Fragmenten. PhD thesis, Lehrstuhl II für Mathematik der Universität Bayreuth (1994). **[cito:extends] [cito:usesMethodIn]**

[2] Mcnaught, A.D., Wilkinson, A.: IUPAC. Compendium of Chemical Terminology, 2nd Ed. (the “Gold Book”), p. 951. Blackwell Scientific Publications, Oxford (1997)

[3] Bóna, M.: Combinatorics of Permutations, 2nd edn. Chapman and Hall/CRC, New York (2012)

[4] Faulon, J.-L., Bender, A.: Handbook of Chemoinformatics Algorithms. Chapman & Hall/CRC, London (2010)

[5] Plewinsky, B., Hennecke, M., Oppermann, W.: Organische verbindungen. In: Das Ingenieurwissen: Chemie, p. 86. Springer, Berlin (2014). Chap. 11

[6] Badertscher, M., Bischofberger, K., Munk, M.E., Pretsch, E.: A novel formalism to characterize the degree of unsaturation of organic molecules. J. Chem. Inf. Comput. Sci. **41**, 889–893 (2001)

[7] Bhattacharya, P.: The representation of permutations by trees. Computers Math. Applic. **28**, 67–71 (1994)

[8] ETH Zürich Euler VII Cluster. `https://scicomp.ethz.ch/wiki/Euler#Euler_VII_.E2.80.94_phase_2`. Accessed: 13.09.2022

[9] Weininger, D., Weininger, A., Weininger, J.L.: SMILES. 2. Algorithm for generation of unique SMILES notation. J. Chem. Inf. Comput. Sci. **29**, 97–101 (1989). **[cito:usesMethodIn]**

[10] Schneider, N., Sayle, R.A., Landrum, G.A.: Get your atoms in order. An open-source implementation of a novel and robust molecular canonicalization algorithm. J. Chem. Inf. Model. **55**, 2111–2120 (2015). **[cito:usesMethodIn]**
